# Imprecise Cas12a/ssODN‐Mediated Editing of *eIF4E1* Confers Dominant‐Negative Resistance to Potato Virus Y in *Solanum tuberosum*

**DOI:** 10.1111/mpp.70305

**Published:** 2026-06-30

**Authors:** Alessandra Lucioli, Raffaela Tavazza, Attila Molnar, Vincenza Ilardi, Adéla Přibylová, Mario Tavazza

**Affiliations:** ^1^ Division Biotechnologies, ENEA—Italian National Agency for New Technologies Energy and Sustainable Economic Development, Casaccia Research Center Rome Italy; ^2^ Institute of Molecular Plant Sciences University of Edinburgh Edinburgh UK; ^3^ Research Centre for Plant Protection and Certification (CREA‐DC) Council for Agricultural Research and Economics (CREA) Rome Italy; ^4^ Department of Experimental Plant Biology, Faculty of Science Charles University Prague Czech Republic

## Abstract

Mutants in the eukaryotic initiation factor 4E (*eIF4E*) gene family provide recessive resistance to plant viruses. Previously, we demonstrated that knocking out *SteIF4E1* potato alleles (*A, B, B, B1*) resulted in limited resistance to a necrogenic PVY isolate (NTN). Expanding on this, we used a non‐transgenic method employing Cas12a and a single‐stranded oligodeoxynucleotide (ssODN) as a repair template to introduce amino acid changes in SteIF4E1, attempting to mimic the *Capsicum* PVY resistance allele *pvr2* ^*1*^. However, no plants that had been regenerated from transfected protoplast cells displayed correct *SteIF4E1* editing. Instead, truncated sequence duplications (pvr2^1^SD) appeared at some mutated alleles, suggesting that ssODN‐mediated repair predominantly occurs via microhomology‐mediated end joining. Importantly, out of the 39 edited lines tested, the dwarf line Bb29 exhibited resistance to PVY‐NTN and showed reduced systemic infection sustained by PVY‐O. Notably, PVY‐NTN resistance‐breaking isolates occasionally emerged in Bb29, confirming genuine eIF4E‐mediated resistance. In Bb29, three of four *eIF4E1* copies were mutated (*AΔ12, BΔ6, B, B1pvr2* ^*1*^ *SD*), with *SteIF4E1_B1pvr2* ^*1*^ *SD* featuring a premature stop codon at the 5′ end of the mutated sequence, and the unique *SteIF4E1_AΔ12* allele in our gene‐edited lines. We found that *SteIF4E1_BΔ6*, but not *SteIF4E1_AΔ12*, partially complemented an eIF4E‐deficient yeast strain. In addition, introducing a wild‐type copy of *SteIF4E1* into Bb29 restored virus susceptibility and partially reversed dwarfism. Conversely, transforming wild‐type potatoes with *SteIF4E1_AΔ12* mimicked the resistance profile of Bb29, conferring stronger and broader resistance against PVY than the *SteIF4E1* knockout approach. These findings reveal a dominant‐negative resistance mechanism, opening new avenues for engineering potyvirus resistance.

## Graphical abstract

Efforts to mimic the pepper PVY‐resistant *pvr2* ^*1*^ allele in potato using Cas12a‐mediated single‐stranded oligodeoxynucleotide (ssODN) editing of *eIF4E1*, while highlighting key technical limitations, shed light on a dominant‐negative PVY resistance mechanism. This involves the *eIF4E1_AΔ12* allele, which confers broader and stronger resistance than *eIF4E1* knockout.

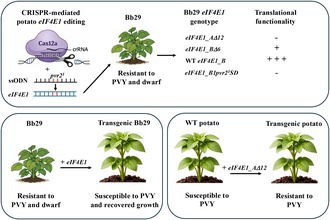

## 1 Introduction

Potato ( *Solanum tuberosum* ) is the fourth most important food crop globally and a key source of starch, proteins and vitamins. However, as a vegetatively propagated species, it is highly susceptible to pathogens, particularly *Potato virus Y* (PVY), the most destructive virus of potato worldwide. PVY, the type member of the *Potyvirus* genus, can reduce yields by 10%–85% depending on cultivar, virus strain and environmental conditions (Valkonen 1997, 2007). Some strains, such as PVY‐NTN, cause tuber necrosis and severe quality losses (Lacomme and Jacquot 2017). The virus is transmitted non‐persistently by more than 60 aphid species (Lacomme and Jacquot 2017), and because insecticides fail to block this mode of transmission (Kirchner et al. 2014), genetic resistance remains the most effective control strategy.

The study of susceptibility (*S*) genes—host factors required for pathogen infection—has gained renewed attention with recent advances in genome editing. *S* gene‐based resistance is recessive, requiring loss‐of‐function mutations in all alleles to be effective. Among known *S* genes, members of the eukaryotic translation initiation factor 4E (eIF4E) family are essential for infection by potyviruses, which exploit eIF4E through interactions with the viral VPg protein (Bastet et al. 2017; Truniger and Aranda 2009).

In flowering plants, the eIF4E family includes eIF4E, eIF(iso)4E and nCBP; only the first two function in canonical translation (Browning and Bailey‐Serres 2015; Patrick and Browning 2012). In tomato, pepper and potato, this family is encoded by four genes (*eIF4E1*, *eIF4E2*, *eIF(iso)4E* and *nCBP*). Naturally occurring *eIF4E* alleles conferring potyvirus resistance have been reported in pepper, tobacco and tomato, but not in cultivated potato (Ibiza et al. 2010; Julio et al. 2015; Ruffel et al. 2002, 2005). In pepper, polymorphisms are clustered within two regions around the cap‐binding pocket (regions I and II). These mutations strongly affect the electrostatic potential and the binding of VPg, a multifunctional PVY protein that is covalently attached to the 5′ end of the viral RNA and acts as a protein cap, leading to distinct resistance spectra (Charron et al. 2008; Poulicard et al. 2016). Mutations in VPg often enable resistance breakdown (Charron et al. 2008; Poulicard et al. 2016; Ruffel et al. 2005). In potato, most single amino acid substitutions in the eIF4E1 region I reduce its interaction with VPg (Lebedeva et al. 2024).

Functional studies in solanaceous crops have validated eIF4E genes as strategic targets for virus resistance. Mutagenesis of *eIF4E1* or *eIF4E2* in tomato or tobacco confers strain‐specific immunity to several potyviruses (Julio et al. 2015; Moury et al. 2020; Piron et al. 2010; Zhao et al. 2020). Similarly, RNA interference targeting eIF4E transcripts confers PVY resistance (Mazier et al. 2011; Miroshnichenko et al. 2020, 2025; Takakura et al. 2018). The introduction of RNA‐guided nucleases, including CRISPR‐Cas9 (Jinek et al. 2012) and Cas12a (Zetsche et al. 2015), has accelerated targeted eIF4E disruption through double‐strand DNA breaks repaired mainly by non‐homologous end joining (NHEJ) (Přibylová and Fischer 2024). Gene knockouts generated via this approach have conferred potyvirus resistance in *Arabidopsis*, tomato, pepper and potato (Kumar et al. 2022; Kuroiwa et al. 2022; Le et al. 2022; Liu et al. 2025; Lucioli et al. 2022; Pyott et al. 2016; Noureen et al. 2022; Yoon et al. 2020). To broaden resistance to multiple PVY strains and other tomato‐infecting potyviruses, researchers have pursued double‐null mutations targeting both *eIF4E1* and *eIF4E2* using EMS mutagenesis or CRISPR‐Cas approaches (Gauffier et al. 2016; Kumar et al. 2022). Notably, simultaneous null mutations in *eIF4E1* and *eIF4E2* confer broader potyvirus resistance (Gauffier et al. 2016), but often at the cost of impaired plant development (Gauffier et al. 2016; Kumar et al. 2022).

As non‐templated edits rarely recover gene function or produce purposeful changes beyond knockout, a more refined editing strategy is required. To promote precise knock‐in/templated repair, a DNA template containing extended homologous sequences flanking the Cas‐generated double‐strand break (DSB) can be provided. These homologous arms are recognized by the cell's native DNA repair systems, which naturally use homology around the DSB to guide repair (Van Vu et al. 2025). As such template, single‐stranded oligodeoxynucleotides (ssODNs) can be used. This technique, referred to as single‐strand templated repair (SSTR), is considered one of the most efficient precision DNA repair methods in eukaryotes, including human cell lines (Chen et al. 2011; Richardson et al. 2018) and algae (Ferenczi et al. 2017). SSTR has been tested in both protoplasts (Jiang et al. 2021; González et al. 2025) and plants (Sauer et al. 2016; Lu et al. 2020); however, its application is currently limited to Cas9.

Recently, an iterative approach combining TALEN‐based gene knock‐in and cytosine base editors was used to introduce non‐synonymous mutations into the tomato *eIF4E1* gene (Kuroiwa et al. 2023). The mutation in the region I does not confer resistance, whereas some combinations in region II associated with the mutation in the region I preserved eIF4E1 function while extending the resistance spectrum beyond that of simple knockout lines, while others mimicked the PVY resistance seen in *eIF4E1* null mutants, showing the complexity of balancing eIF4E function and virus resistance (Kuroiwa et al. 2023).

In potato, CRISPR‐Cas9 knockout of *eIF4E1* in cultivar Désirée conferred partial resistance to PVY‐NTN, which is evident only when all four gene copies were inactivated (Lucioli et al. 2022). This observation confirmed the recessive nature of *S*‐gene‐based resistance and prompted exploration of more refined editing strategies.

In this study, we used a non‐transgenic strategy to assess whether Cas12a‐mediated SSTR could support precise editing of *eIF4E1* in the potato cultivar Désirée, aiming to mimic the naturally occurring PVY‐resistant *pvr2* ^*1*^ allele in pepper. Protoplasts were transiently transfected with ssODNs serving as repair templates together with Cas12a/crRNA delivered as ribonucleoprotein complexes or plasmid constructs. The resulting lines exhibited diverse *eIF4E1* mutations, including small deletions and knock‐ins, but no perfect template‐guided edits, suggesting that microhomology‐mediated end joining (MMEJ), also referred to as polymerase theta‐mediated end joining, was the predominant repair pathway (Přibylová and Fischer 2024). Remarkably, one edited line (Bb29) showed strong resistance to multiple PVY‐NTN isolates and partial resistance to PVY‐O, despite carrying mutations in only three of the four *eIF4E1* alleles. Molecular analyses revealed a dominant‐negative mechanism underlying this resistance, establishing a novel paradigm for engineering virus resistance by modifying an *S*‐gene.

## 2 Results

### 2.1 *pvr2* ^*1*^‐Like Mutations Affect the Function of Potato *eIF4E1* *A* and *B* Alleles Differently

**FIGURE 1:**
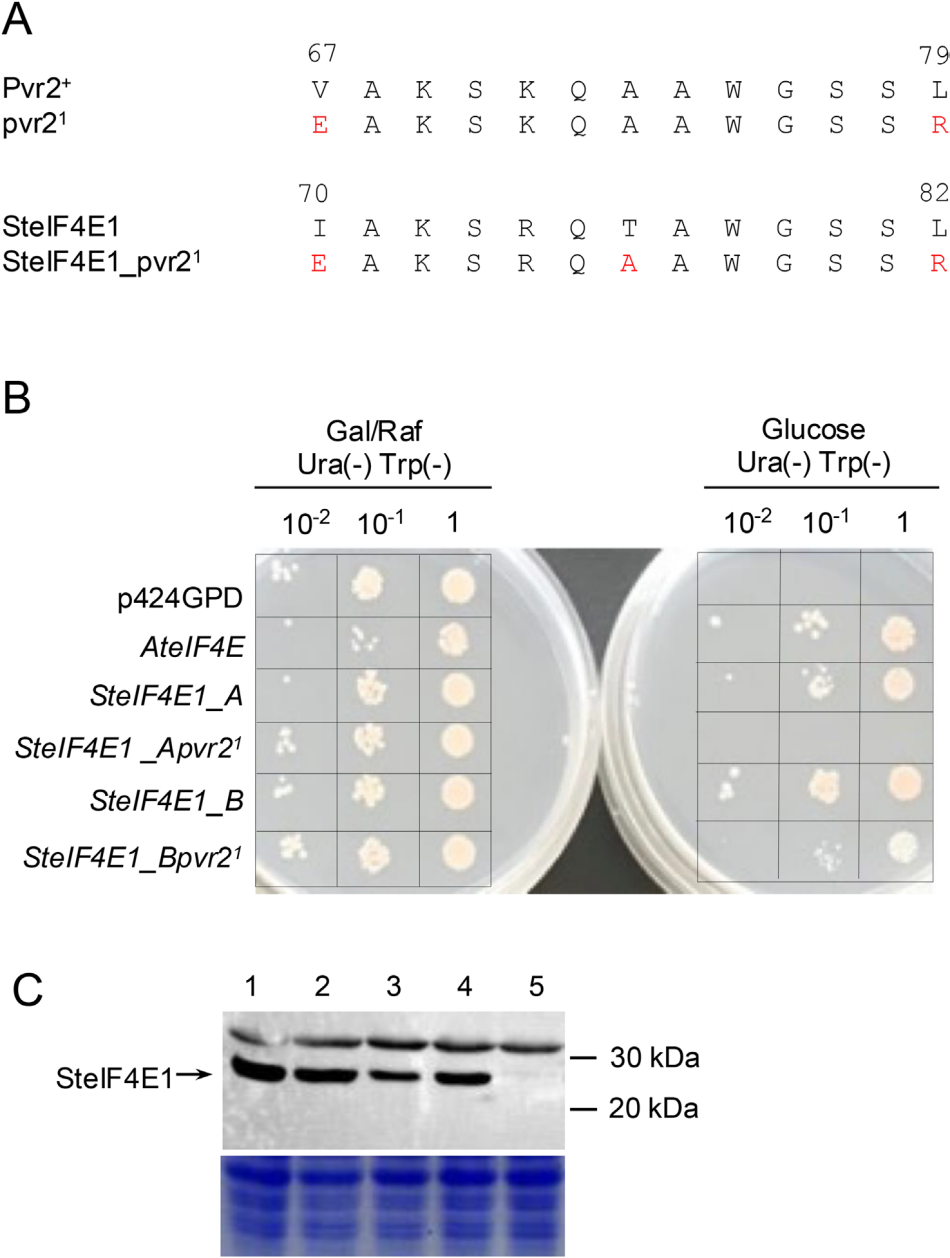
Mutagenesis strategy of the *Solanum tuberosum* ‘Désirée’ SteIF4E1 protein and complementation of yeast strain JO55 with wild‐type and mutated *SteIF4E1* cDNAs. (A) Amino acid sequences of the region I of pepper and potato eIF4E1 proteins. Pvr2^+^, pepper eIF4E1 PVY‐susceptible form; pvr2^1^, pepper eIF4E1 PVY‐resistant form; SteIF4E1, wild‐type eIF4E1 potato protein; SteIF4E1_pvr2^1^, potato eIF4E1 mimicking pvr2^1^. Mutated amino acids in pepper and potato sequences are marked in red. (B) The yeast strain JO55 was transformed with either an empty p424GPD plasmid (negative control) or p424GPD constructs expressing *AteIF4E* (positive control) or potato wild‐type and mutated *SteIF4E1* A and B alleles. Dilutions were spotted on galactose/raffinose (Gal/Raf) synthetic dropout medium lacking uracil and tryptophan, and on a selective nutrient dropout medium containing glucose and lacking uracil and tryptophan. Only yeast functionally complemented by the ectopic expression of an eIF4E protein can grow on the selective medium. (C) Western blot analysis of potato SteIF4E1 proteins expressed in yeast strain JO55. (1) SteIF4E1_Apvr2^1^, (2) SteIF4E1_Bpvr2^1^, (3) SteIF4E1_A, (4) SteIF4E1_B, (5) p424GPD. The arrow identifies the potato eIF4E1 protein (26 kDa). All strains were grown on galactose and raffinose, and a western blot was done using anti‐NteIF4E1 antibodies (Combe et al. 2005). Lower panel, Coomassie staining of a replica gel to check protein loading.

Natural *eIF4E* resistance alleles often provide stronger potyvirus resistance than null mutations (Bastet et al. 2017). In pepper, PVY‐resistance–associated substitutions cluster within protein domains I and II (Ibiza et al. 2010; Poulicard et al. 2016; Rubio et al. 2009; Ruffel et al. 2002). These domains correspond to exons 1 and 2 in both pepper and potato *eIF4E*. The pepper *pvr2* ^*1*^ allele carries two substitutions (V67E and L79R) relative to the susceptible *Pvr2* ^*+*^. To test whether mimicking *pvr2* ^*1*^ confers PVY resistance in potato, we first characterized the *SteIF4E1* alleles in cv. Désirée, identifying three variants A, B and B1, with two copies of the B allele (Figure S1). The eIF4E1_A protein is identical to potato cv. Russet Burbank AEW07371.1, while eIF4E1_B and eIF4E1_B1 differ by eight and six residues, respectively (Figure S2). Alignment revealed that pepper V67 and L79 correspond to I70 and L82 in *SteIF4E1* (Figure 1A), a region fully conserved among the three potato alleles (Figure S2). Compared to *pvr2^1^*, the potato sequence also contains K74R and A76T substitutions. However, the K74R substitution was not considered further because this residue is conserved between the susceptible *Pvr1* ^*+*^ and resistant *pvr2* ^*1*^ alleles in pepper (Rommens et al. 2015). To assess the impact of mimicking the *pvr2* ^*1*^ allele on protein translation, we introduced I70E, T76A and L82R substitutions into the potato *eIF4E1 A* and *B* alleles (Figure 1A), referred to as *SteIF4E1_Apvr2* ^*1*^ and *SteIF4E1_Bpvr2* ^*1*^, respectively, and then performed a yeast complementation assay (Figure 1B). *SteIF4E1_Bpvr2* ^*1*^ supported growth under selective conditions but less efficiently than the corresponding controls, whereas *SteIF4E1_Apvr2* ^*1*^ failed to complement despite normal protein accumulation (Figure 1B,C). Introducing only two substitutions I70E and L82R into *SteIF4E1_A* did not preserve its function either (Figure S3), indicating that the *pvr2* ^*1*^‐like mutations affect the function of potato *eIF4E1 A* and *B* alleles differently.

### 2.2 Cas12a/ssODN Induces Aberrant *SteIF4E1* Alleles

**FIGURE 2:**
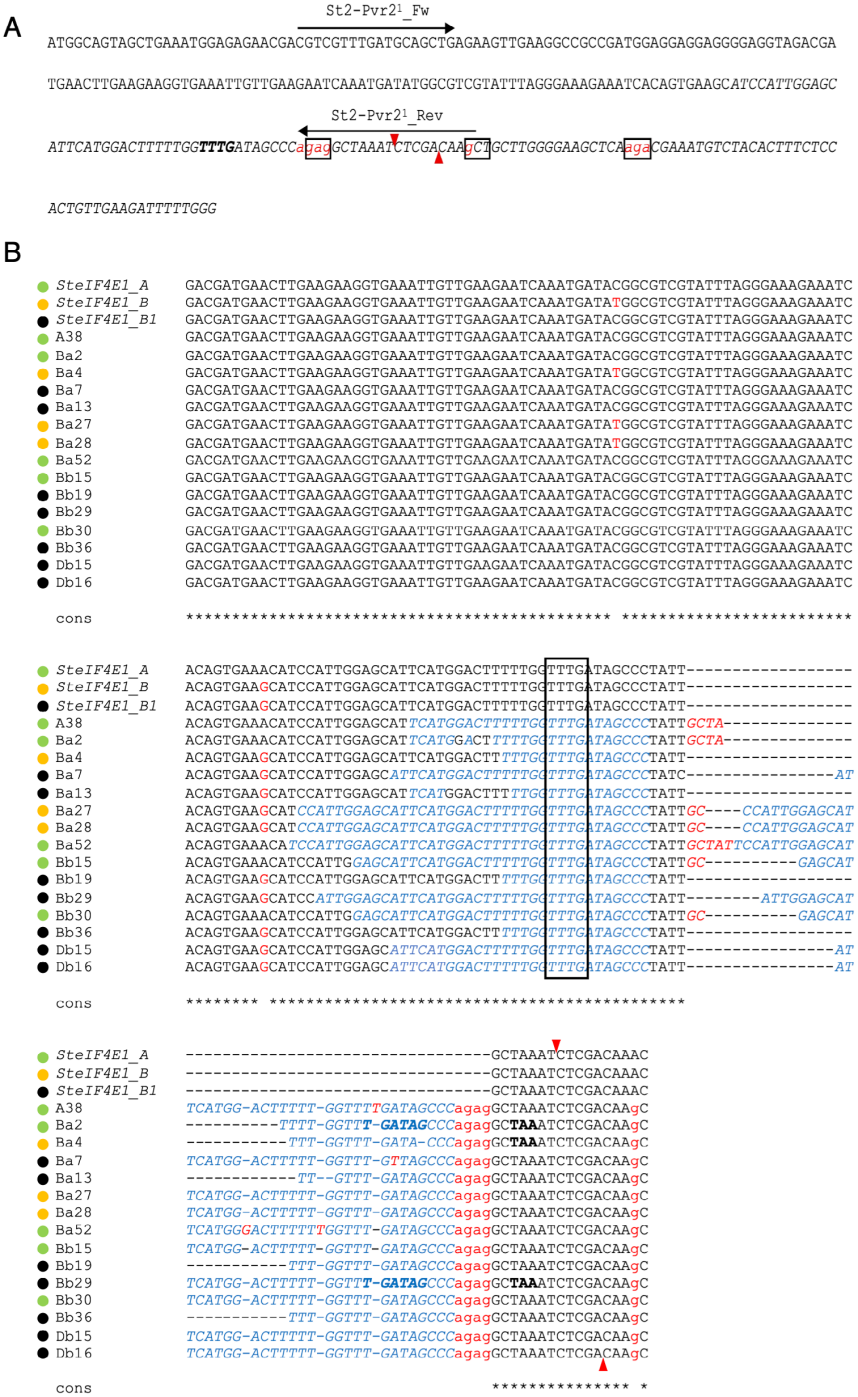
Sequencing Cas12a‐edited potato plants identifies the presence of duplicated sequences at the 5′ of the introduced mutations. (A) Expected nucleotide sequence of the first exon of the *SteIF4E1_B* allele based on the Cas12a‐editing strategy. Black arrows at the top of the sequence identify the forward and reverse primers used to screen protoplast‐derived plants. In italics, the sequence region used as ssODN for Cas12a‐mediated editing. Red lowercase letters identify mutated nucleotides mimicking *pvr2* ^*1*^. Boxed sequences identify triplets encoding the mutated amino acids. Red arrows indicate the cut sites of Cas12a on the double‐stranded DNA at 18 and 23 nucleotides from the PAM site. (B) Sequences of the PCR fragments amplified from Cas12a‐edited plants with the primers shown in panel A. Single‐nucleotide polymorphism, red uppercase letters. Duplicated nucleotide sequences, light blue and italic. Extra added nucleotides, italic red uppercase letters. Nucleotide deletions, red em dashes. Stop codons, bold letters. Boxed sequences indicate the Cas12a PAM site. Red arrows indicate the cut sites of Cas12a on the double‐stranded DNA at 18 and 23 nucleotides from the PAM site. Green, orange and black dots identify *SteIF4E1_A*, *SteIF4E1_B* and *SteIF4E1_B1* alleles, respectively.

To introduce *pvr2* ^*1*^‐like amino acid substitutions into *SteIF4E1*, we used a non‐transgenic protoplast transfection approach combining a 120‐nt ssODN donor (40‐nt homology arms) with Cas12a/crRNA delivered either as a ribonucleoprotein complex or as a plasmid (Figure 2A). The Cas12a target site was selected that specifically recognized *SteIF4E1* but not *SteIF4E2 or SteIF(iso)4E* (Figure S4). Five independent experiments (A, Ba, Bb, Da, Db) generated 633 regenerated plants, 45 of which (7.1%) tested positive in a PCR screening using a forward oligonucleotide conserved among all *SteIF4E1* alleles and a reverse oligonucleotide complementary to the mutagenized sequences (Table S1). Sequencing of 16 randomly selected edited lines revealed that all carried duplicated sequences at the 5′ end of the mutagenized region, 15 of which contained a single edited allele (Figure 2B), while one, Ba44 (not shown), carried both A‐ and B‐mutated alleles. To verify whether the presence of duplicated sequences reflected a general phenomenon, we subjected all 45 potato lines to a PCR screening that, in the case of correct integration of the mutated ssODN, should amplify only one 110‐bp fragment in both wild‐type (WT) and mutated *SteIF4E1_ pvr2* ^*1*^ alleles. PCR assays of all 45 plants consistently detected additional, slower‐migrating fragments besides the expected 110‐bp product (Figure S5), indicating sequence duplications (pvr2^1^SD). These results show that Cas12a/ssODN targeting of *SteIF4E1* in protoplasts triggers inefficient, aberrant homology‐mediated mutagenesis, regardless of whether Cas12a was delivered as a ribonucleoprotein (transfection experiments A, Ba and Bb) or via plasmid expression (transfection experiments Da and Db).

### 2.3 The Bb29 Line Shows Resistance to Necrogenic PVY Isolates and Enhanced Resistance to PVY‐O

**FIGURE 3:**
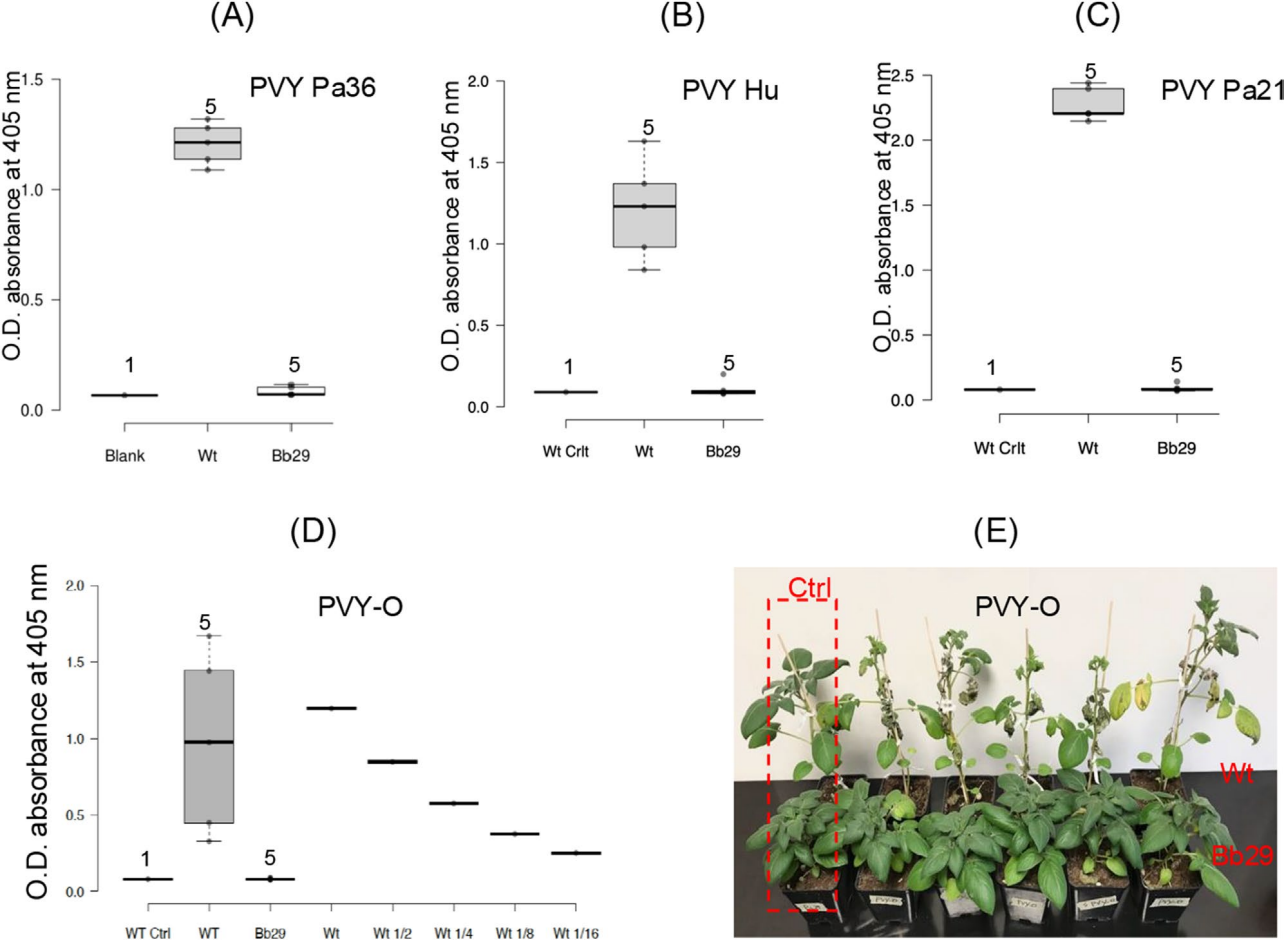
The Bb29 line shows resistance to necrogenic PVY isolates and enhanced resistance to PVY‐O. Virus accumulation at 20, 21 and 14 days post‐inoculation (dpi) of the necrogenic isolates PVY‐Pa36 (panel A), PVY‐Hu (panel B) and PVY‐Pa21 (panel C), respectively. PVY‐O accumulation in the uppermost non‐inoculated leaves at 14 dpi (panel D) and the phenotype of PVY‐O challenged plants at 18 dpi (panel E). Virus accumulation was evaluated by double‐antibody sandwich‐ELISA. For PVY‐O, a pool of extracts from the five wild‐type plants was used in a two‐fold dilution series to assess virus accumulation. Ctrl, uninoculated control plants; Wt, wild‐type plants. Centre lines show the medians; box limits indicate the 25th and 75th percentiles, as determined by R. The numbers above the boxes indicate the number of plants analysed.

To test whether ssODN‐mediated edits or indels at *SteIF4E1* conferred PVY resistance, three clones of each edited line capable of rooting (39/45) were challenged with a necrogenic isolate (NTN) of PVY (PVY‐Pa36). Virus resistance was assessed between 21 and 30 days post‐inoculation (dpi) using a double‐antibody sandwich (DAS)‐ELISA. All 39 edited potato lines, except line Bb29, which exhibited a dwarf phenotype, were susceptible to PVY‐Pa36 (Figure S6). In three independent experiments, Bb29 exhibited strong resistance to PVY‐Pa36, PVY‐Hu and PVY‐Pa21 (Figure 3A–C). The cultivar Désirée carries the dominant *Ny* gene, which confers hypersensitive response (HR)‐based resistance to PVY‐O (Funke et al. 2017; Jones and Vincent 2018; Kehoe and Jones 2016). As the PVY‐O isolate used induces systemic HR in WT Désirée, we compared responses in Bb29. Local HR developed similarly in both genotypes (Figure S7), but only WT plants showed systemic necrosis and high viral accumulation in upper leaves (14–18 dpi), whereas Bb29 displayed minimal necrosis and strongly reduced PVY‐O accumulation (Figure 3D,E; Figure S8). This evidence suggests that Bb29 possesses a mechanism capable of conferring high resistance to three necrogenic PVY isolates, while simultaneously mitigating the systemic HR triggered by PVY‐O.

### 2.4 Resistance‐Breaking Isolates Can Emerge in Bb29 Plants Challenged With PVY‐Pa36

**FIGURE 4:**
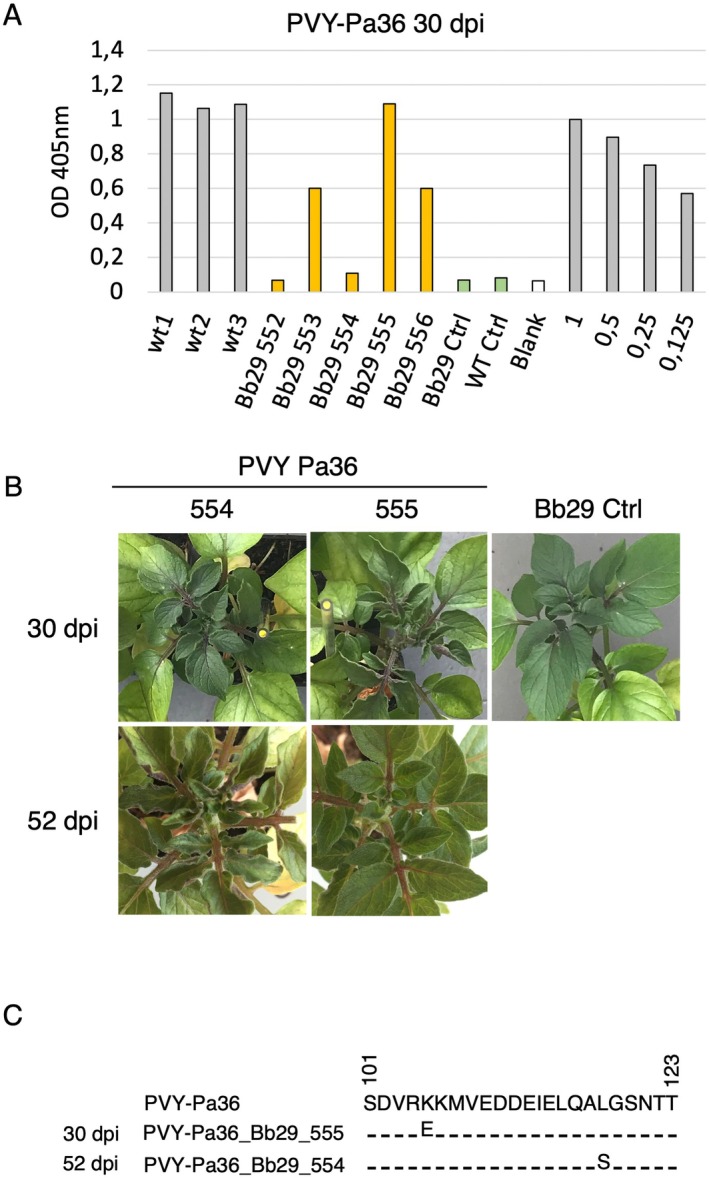
Viral mutants can emerge in Bb29 plants challenged with PVY‐Pa36. (A) Virus accumulation at 30 days post‐inoculation (dpi) was assessed by double‐antibody sandwich‐ELISA. The sap from the three PVY‐Pa36 infected wild‐type plants was mixed and used for generating the standard dilution curve. (B) Virus symptoms at 30 and 52 dpi on Bb29 554 and 555 plants. (C) Amino acid sequence of the central region of the VPg of PVY‐Pa36 and the resistance‐breaking viruses identified in Bb29 555 and 554 plants at 30 and 52 dpi, respectively.

Natural and engineered eIF4E‐based resistances can be overcome by potyviral evolution, often through mutations in the VPg central region enabling interaction with alternative eIF4E isoforms. To test whether PVY‐Pa36 could adapt to Bb29, five resistant plants were inoculated with a high viral load and monitored for 52 days. By 30 dpi, three plants showed infection, with line 555 displaying virus accumulation and a strong infection phenotype (Figure 4A,B). At 52 dpi, plant 554 also became infected. Sequencing of the VPg region revealed distinct point mutations in each plant, K105E in 555 and L115S in 554 (Figure 4C), which restored viral infectivity. Coherently, Bb29 plants inoculated with the K105E isolate became fully susceptible (Figure S9). Mutations at these positions have been linked to *eIF4E* resistance‐breaking in other potyviruses (Janzac et al. 2014; Masuta et al. 1999; Takakura et al. 2018). These results indicate that Bb29's resistance to PVY‐Pa36 relies on an altered eIF4E1 function that can be bypassed, under high virus load and strong selection pressure, by VPg adaptation. To our knowledge, no PVY potato isolates have been reported to possess VPg with the K105E or L115S mutations. This is the first report of PVY overcoming eIF4E‐mediated resistance in potato through VPg‐mediated viral evolution.

### 2.5 The Bb29‐Resistant Line Possesses One Wild‐Type and Three Mutated *eIF4E1* Alleles

**FIGURE 5:**
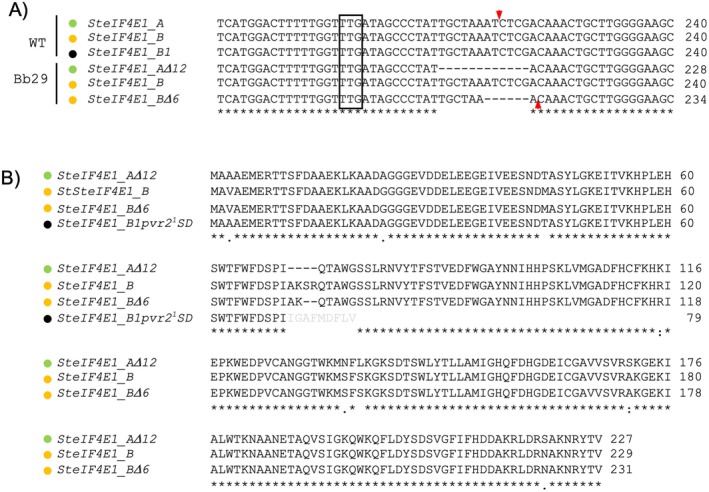
The Bb29 edited potato line possesses one wild‐type *SteIF4E1_B* gene. (A) Alignment of sequences around the Cas12a cut site of *SteIF4E1_A*, *SteIF4E1_B* and *SteIF4E1_B1* alleles with the eIF4E1 cDNA sequences from the Bb29 plant line. Boxed sequences indicate the Cas12a PAM site. Red arrows indicate the cut sites of Cas12a on the double‐stranded DNA at 18 and 23 nucleotides from the PAM site. (B) Alignment of the predicted amino acid sequences encoded by the Bb29 *eIF4E1* alleles. The allele *SteIF4E1_B1pvr2^1^SD* can potentially encode a truncated protein of 70 plus 9 extra amino acids (light grey) generated by the presence of out‐of‐frame duplicated sequences. Green, orange and black dots identify *SteIF4E1_A*, *SteIF4E1_B* and *SteIF4E1_B1* alleles, respectively.

To determine the *SteIF4E1* genotype of Bb29, full‐length cDNAs were amplified and sequenced, revealing three alleles: A and two copies of B. Sequence analysis of these cDNAs uncovered that Cas12a‐mediated editing resulted in a 12‐nt deletion in the A allele (*SteIF4E1_AΔ12*) and a 6‐nt deletion in one of the B alleles (*SteIF4E1_BΔ6*), both leading to in‐frame short deletions. The second B allele remained wild‐type (Figure 5). Intriguingly, no B1 allele‐specific cDNA sequences were recovered. This was unexpected, given that our previous genomic DNA analysis (Figure 2) identified an ssODN‐derived sequence duplication at the B1 allele upstream of the Cas12a cleavage site. Subsequent PCR attempts to amplify intact 3′ B1 sequences from the Bb29 genomic DNA failed. We then conducted inverse PCR using primers specifically designed to anneal to the B1 allele. However, this approach only amplified the B allele, but not the B1 allele (Figure S10), suggesting that a DNA structure or rearrangement at the 3′ end of the mutated B1 allele could inhibit DNA synthesis and the amplification of the full‐length B1 locus. Notably, the truncated allele, which we refer to as Bb29 *SteIF4E1_B1pvr2* ^*1*^ *SD* harbours a premature double stop codon. This could only encode a small truncated protein (Figure 5B) lacking domains essential for Cap, VPg and eIF4G binding, as described in previous studies (Miras et al. 2017; Lebedeva et al. 2024; Monzingo et al. 2007). Furthermore, yeast complementation assays demonstrated that *SteIF4E1_BΔ6* retained partial function, whereas *SteIF4E1_AΔ12* was nonfunctional (Figure S11). Thus, Bb29 carries one fully functional wild‐type allele (B), and one partially functional allele (BΔ6) eIF4E1_B protein.

### 2.6 Bb29 Exhibits a Unique Genotype Among the Edited Potato Lines

**FIGURE 6:**
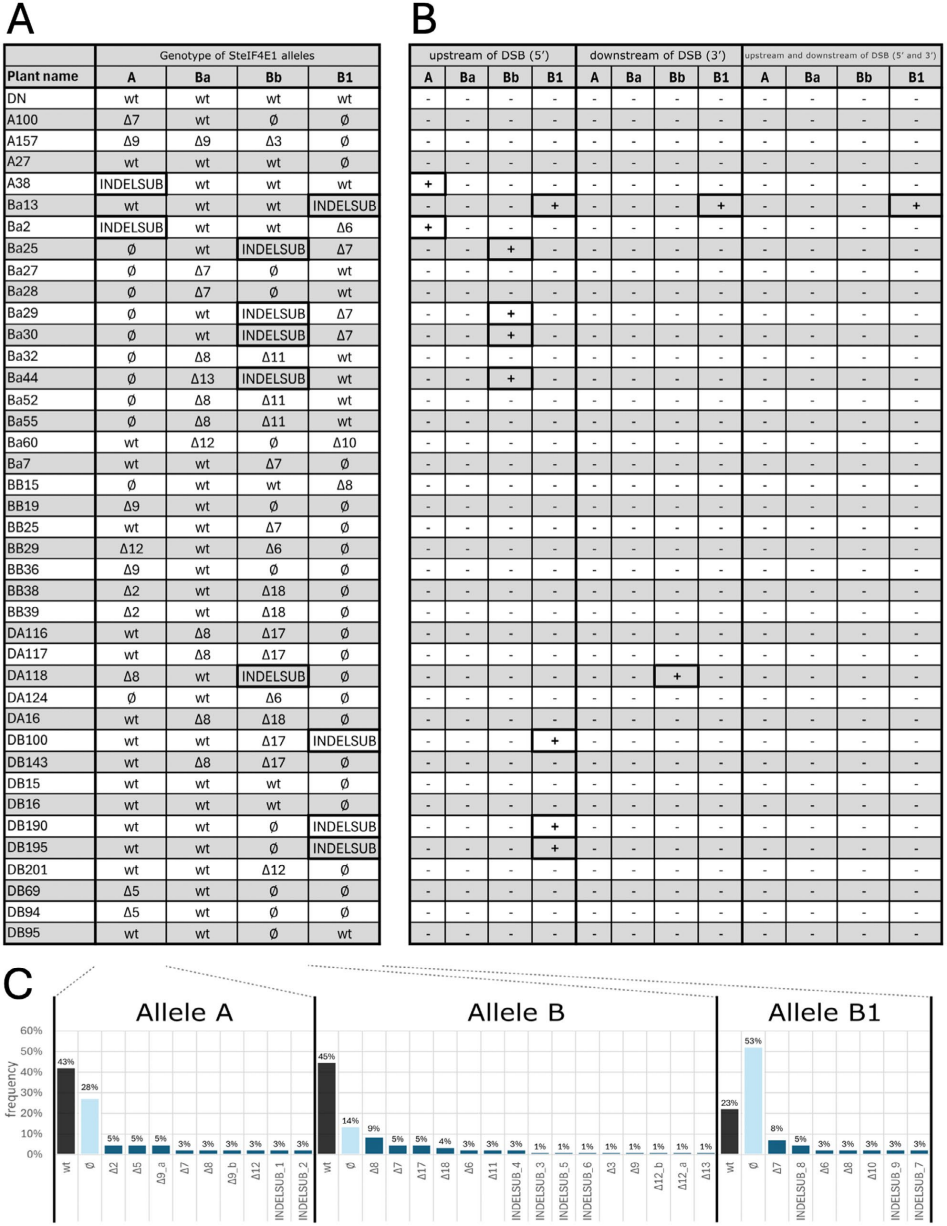
Amplicon‐based targeted‐sequencing of *SteIF4E1* alleles of the edited potato lines. (A) Characterization of mutations in each *SteIF4E1* allele in the regenerated plants by targeted amplicon sequencing. (B) Analysis of the presence of ssODN‐mediated substitutions upstream (GCCCagagGCTA) and downstream (GCTCAagaCGAA) of the Cas12a‐induced double‐stranded break (DSB) and the entire 5′ to 3′ (agagGCTAAATCTCGACAAgCTGCTTGGGGAAGCTCAaga) sequence for each allele. ‘+’ and ‘−’ indicate the presence or absence of the sequence, respectively. (C) Summary of the genotypes and corresponding mutation frequencies of each *SteIF4E1* allele in the gene‐edited lines. For allele B, we were unable to determine which copy the mutation belongs to, as the two copies share the same sequence; thus, alleles *Ba* and *Bb* can be interchanged. wt = allele without mutation; Δ = deletion (Δ5 = deletion of 5 nt); ∅ = empty set (allele was not detected in amplicon seq); INDELSUB = a combination of insertion, deletion and substitution events within the allele (_1, _2, … different combinations). In each sample, only one combination was identified. The number of reads can be found in Table S2.

Targeted amplicon sequencing of the *SteIF4E1* edited region in all 39 lines revealed distinct mutation patterns across alleles A, B and B1. Sequencing reads were mapped to reference alleles using diagnostic SNPs, and CRISPResso2 analysis identified insertions, deletions, substitutions and ssODN‐mediated events at base‐pair resolution (Figure 6A). Intriguingly, the most frequent outcome was loss of detectable allele sequences (∅), likely due to rearrangements or secondary structures preventing PCR amplification, as observed for Bb29. This phenomenon affected B1 most often (53%), followed by A (28%) and B (14%). Short deletions (2–18 nt; Δ) represented the next most common class (Figure 6C). Across all lines, most harboured edits in three alleles (59%), followed by two (21%) or one (15%); only line A157 displayed mutations in all gene copies. Notably, the analysis of the 39 genotypes (Figure 6A) showed that no other edited lines have the same genotype as Bb29. In addition, only Bb29 harbours a 12 nt deletion in the A allele. Conversely, the six‐nucleotide deletion in the Bb29 B allele, which removes the amino acids SR (Figure S11A), is identical to the mutated B allele of the PVY‐susceptible Da124 line. Collectively, these findings show that Bb29 has a unique genotype among the 39 edited lines and jointly support a major role for *SteIF4E1_AΔ12* in PVY resistance.

### 2.7 Imprecision of the Cas12a‐Mediated Single‐Strand Templated Repair

To assess the efficacy and accuracy of ssODN‐mediated DNA repair, we mapped the intended 5′ (agag) and 3′ (aga) SNPs/substitutions at the Cas12a cut sites across all genotyped *eIF4E1* alleles in each line (Figure 6B). Eleven lines (28%) harboured substitutions, each affecting a single allele. The overlap between genotypes and ssODN integration patterns in lines Ba25, Ba29 and Ba30, as well as in Db190 and Db195, suggests that these plants are likely clonal. Only one line, Ba13, contained both the 5′ and 3′ SNPs (Figure 6B). Closer inspection of ssODN integration revealed partial sequence duplications spanning the Cas12a cut site in these lines (Figure S12), confirming previous PCR and Sanger sequencing data (Figure 2). Interestingly, in most cases, partial sequence duplications were observed at both ends of the Cas12a DSB. These events were detected in the A, B and B1 alleles of lines A38, Ba13, Ba44, Db100 and Db190 (Figure S12), whereas in line Ba2, sequence duplications were restricted to regions upstream of the Cas12a DSB, and in lines Ba25 and Da118, the duplicated sequences were present in the reverse orientation (Figure S12). The presence of these integration events suggests that ssODNs were not involved in homology‐mediated repair requiring extended DNA pairing, as expected for single‐strand templated repair (SSTR).

### 2.8 Ectopic Expression of Wild‐Type *eIF4E1* in Bb29 Restores PVY Susceptibility and Growth

**FIGURE 7:**
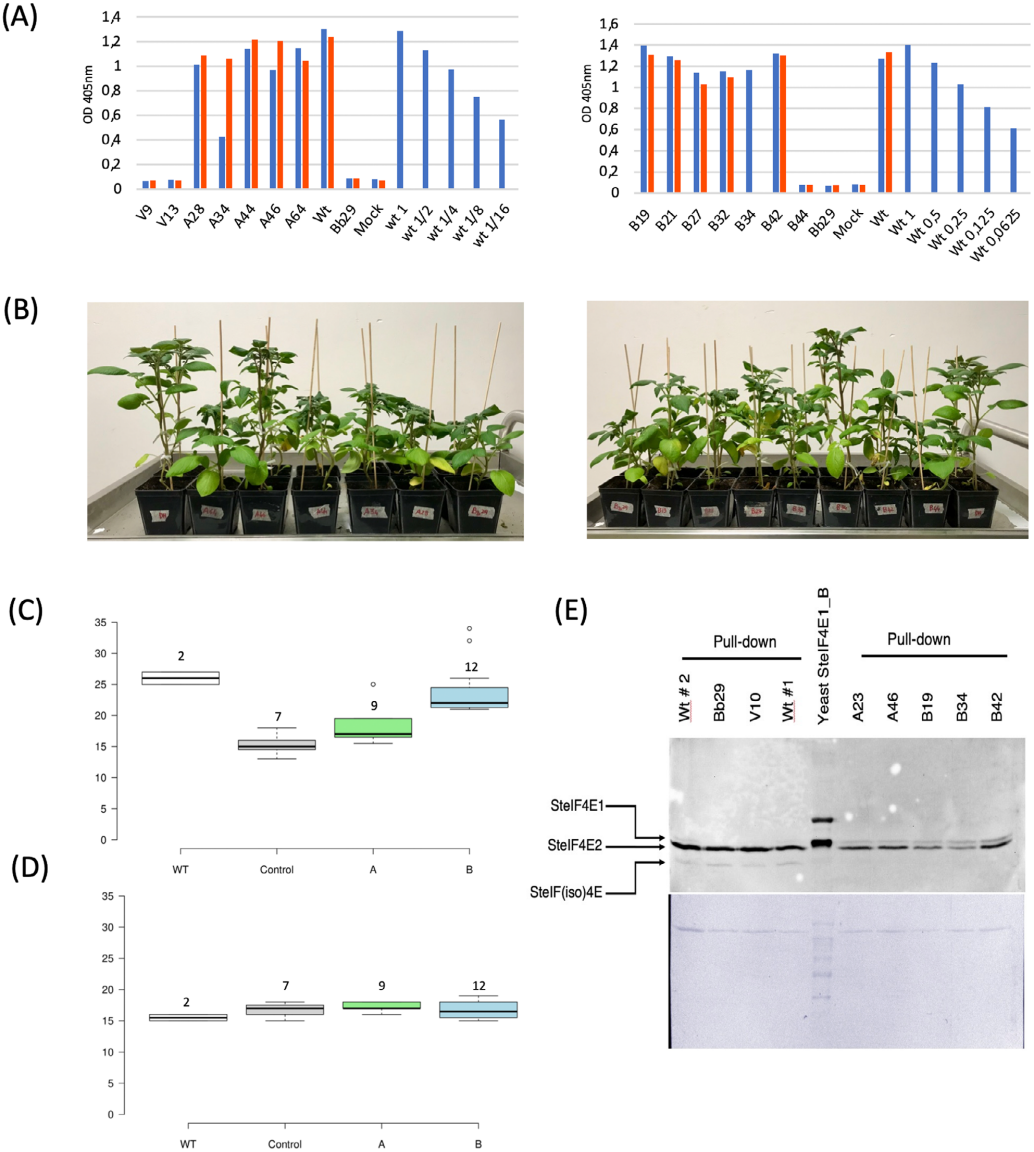
Analyses of potato lines denoted as A, B and V derived from the transformation of the Bb29 plant with the Désirée *SteIF4E1_A*, *SteIF4E1_B* alleles or the empty binary vector, respectively. (A) Virus resistance analysis was conducted at 11 days post‐inoculation (dpi) by measuring virus accumulation via ELISA. At 21 dpi, uninoculated plants were photographed (B), and the height in cm (C) and the number of leaves of each plant class (D) were recorded. Control (Bb29, V9, V13 and the non‐transgenic B44); transgenic A lines (A28, A34, A44, A46 and A64); transgenic B lines (B19, B21, B27, B32, B34 and B42). Centre lines show the medians; box limits indicate the 25th and 75th percentiles, as determined by R. The numbers above the boxes indicate the number of plants analysed. In panel B wild‐type plants are labelled ‘DN’. (E) Western blot analysis of m7GTP pull‐down of leaf protein extracts from wild‐type (wt), Bb29 and transgenic A, B and V potato lines. The yeast SteIF4E1_B is a total protein extract from the yeast strain JO55, which expresses the *SteIF4E1_B* allele (grown in glucose as the carbon source). Wt #1 and Wt #2 are two independent Désirée plants. SteIF4E proteins were detected using rabbit anti‐tobacco eIF4E1‐like antibodies. After antibody detection, the filter was stained with Coomassie brilliant blue to check protein loading (lower panel).

Dwarf phenotypes have previously been observed when both *eIF4E1* and *eIF4E2* genes were downregulated or knocked out (Gauffier et al. 2016; Mazier et al. 2011). To test whether the mutated SteIF4E1 proteins in Bb29 caused both PVY resistance and dwarfing, we transformed Bb29 with the *SteIF4E1_A* or *SteIF4E1_B* allele (A and B lines) or with an empty vector (V lines). In vitro, all transgenic lines resembled Bb29. For each transgenic line, two plants were challenged with PVY‐Pa36, while two were left uninoculated to monitor plant development. At the time of virus inoculation (time zero), the wild‐type plants were taller than plants of A, B and V lines, with no significant height differences among the transgenics (Figure S13A). Following inoculation with PVY‐Pa36, the A and B lines developed viral symptoms and accumulated PVY to wild‐type levels by 11 dpi, while the V lines, B44, which was PCR‐negative for the B wild‐type allele, and Bb29 remained virus‐free (Figure 7A). Uninoculated plants of A and B lines progressively regained height relative to V and Bb29 controls, and by 21 days, some (A46, B34) reached wild‐type stature, which was not linked to an increased number of leaves (Figure 7B–D; Figure S13). Two additional independent experiments, one focused on testing resistance and the other on confirming the ability to recover from dwarfing, yielded similar results (data not shown). Collectively, ectopic expression of eIF4E1 driven by the 35S promoter restored both PVY susceptibility and growth. Thus, the mutated eIF4E1 proteins, either by themselves or through an altered expression of other eIF4E family members induced by the mutated eIF4E1 proteins, could be responsible for the Bb29 phenotype. To test this hypothesis, we decided to investigate the eIF4E expression levels in the wild‐type, Bb29 and the complemented lines. The m7GTP pull‐down assay, followed by western blot analysis using antibodies directed against the tobacco eIF4E1‐like protein (Combe et al. 2005), revealed weak signals for SteIF4E1 and SteIF(iso)4E, and a strong signal for SteIF4E2 in wild‐type plants. In contrast, the SteIF4E1 band was absent in Bb29, whereas it was clearly detectable in the pull‐downs of A and B transgenic plants (Figure 7E). Interestingly, ectopic expression of wild‐type SteIF4E1 altered the levels of eIF4E isoforms. In contrast, Bb29 plants, apart from SteIF4E1, exhibited eIF4E isoform accumulation similar to wild‐type plants. Thus, the Bb29 phenotype does not appear to result from altered expression levels of eIF4E2 and eIF(iso)4E.

### 2.9 Transgenic *SteIF4E1_AΔ12* Plants Exhibit a Resistance Spectrum Similar to the Bb29 Edited Line

**FIGURE 8:**
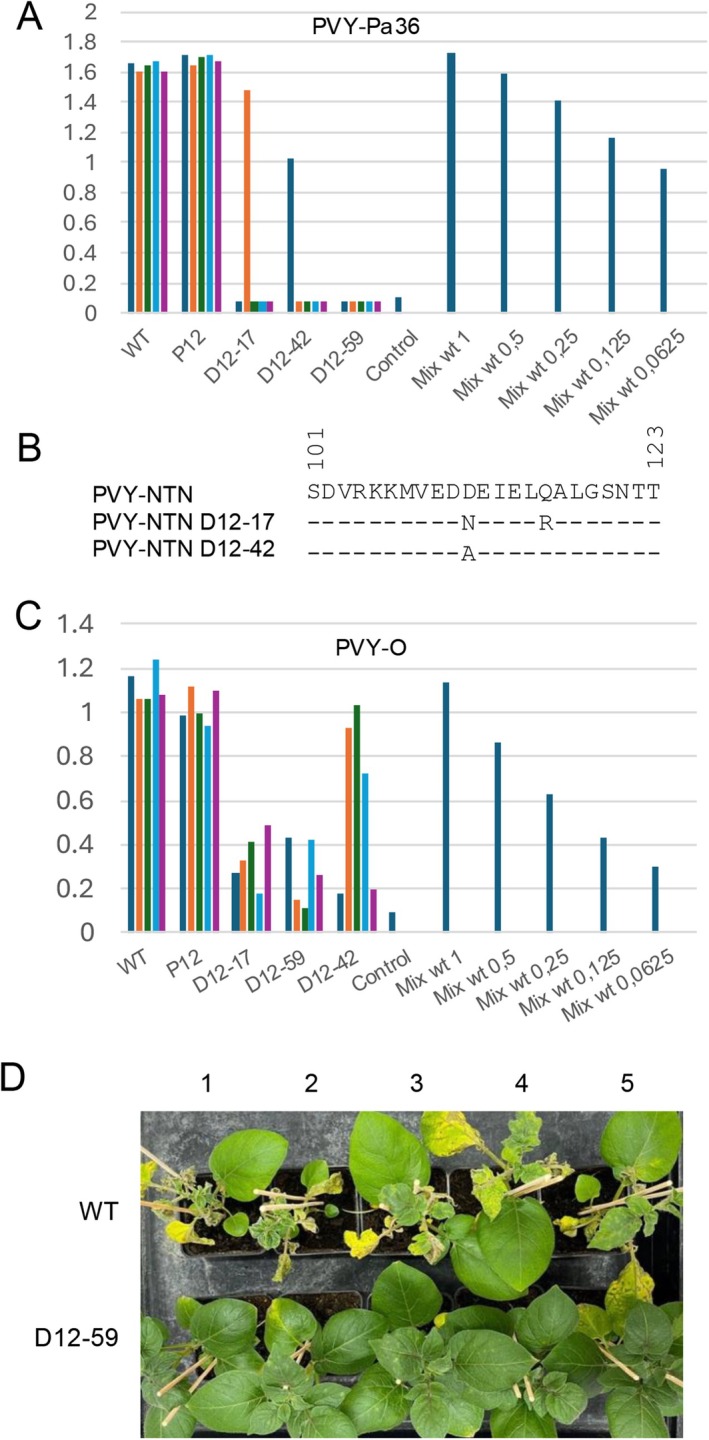
Resistance analyses to PVY of wild‐type (wt) Désirée plants transformed with the Bb29 mutated *eIF4E1_AΔ12* allele. (A) PVY‐Pa36 accumulation was evaluated at 21 days post‐inoculation (dpi) by double‐antibody sandwich ELISA. D12‐17, D12‐42 and D12‐59 are transgenic lines for the *eIF4E1_AΔ12* allele. P12 is a transgenic line for the empty vector. Control is a non‐inoculated wt plant. The sap from the five wt plants was mixed and used to generate the standard curve. (B) VPg sequence of resistance‐breaking isolate accumulating in plant #2 of line D12‐17 and in plant #1 of line D12‐42 at 21 dpi. (C) PVY‐O accumulation at 15 dpi. (D) The phenotype of wt and D12‐59 plants challenged with PVY‐O at 15 dpi.

To collect further evidence for a potential dominant negative mechanism operating in the Bb29 plants, we designed a reverse transgenic approach. We focused on the *SteIF4E1_AΔ12* allele, which was unique among all edited plants, while excluding the partially functional *SteIF4E1_BΔ6* allele that was also present in the susceptible Da124 line (Figure 6). Three plants from each of five independent *SteIF4E1_AΔ* *12* transgenic lines (D12‐4, D12‐17, D12‐42, D12‐59 and D12‐98) and two to three plants from two lines transformed with the empty vector (P7 and P12) were challenged with PVY‐Pa36 and assessed at 30 dpi by DAS‐ELISA. None of the transgenic plants showed virus accumulation (Figure S14). In a repeated experiment involving cuttings from lines D12‐17, D12‐42 and D12‐59, one out of 5 plants from D12‐17 and D12‐42 exhibited moderate PVY‐Pa36 accumulation at 21 dpi, while all other plants remained resistant (Figure 8A). Sequencing of the VPg region indicated that these infections resulted from resistance‐breaking isolates (Figure 8B), as seen earlier in Bb29 plants inoculated with PVY‐Pa36 (Figure 4). Next, we analysed the same lines for their response to PVY‐O. At 15 dpi, all plants were infected, with line D12‐59 performing the best (Figure 8C). Notably, line D12‐59 did not develop systemic necrosis during the period analysed (Figure 8D). Overall, ectopic expression of the *SteIF4E1‐AΔ12* allele closely recapitulated the resistance phenotype observed in Bb29 plants.

## 3 Discussion

Our study demonstrates the generation of a novel PVY resistance in potato through a dominant‐negative modification of eIF4E1 and reveals key constraints of Cas12a‐mediated ssODN knock‐in strategy. Although designed to recreate the PVY‐resistant *pvr2* ^*1*^ allele in potato, the results reveal that imprecise DNA repair governs editing outcomes and shapes virus resistance.

### 3.1 Imprecise Cas12a‐Mediated ssODNs Repair Hinders Precise *eIF4E1* Knock‐In in Potato

In contrast to the precise DNA repair observed in other eukaryotes, such as human cell lines and algae (Chen et al. 2011; Ferenczi et al. 2017; Richardson et al. 2018), our molecular analyses indicate that ssODN‐mediated DNA repair in potato protoplasts predominantly results in imprecise editing, with frequent duplications or rearrangements at the target site. Sanger sequencing of 16 edited plants revealed that all showed duplicated sequences at the 5′ end of the introduced mutations (Figure 2B and Figure S5). In addition, the presence of extra sequences was a general feature in the edited potatoes (Figures S5 and S12). This observation is in line with recent findings using Cas9 to edit the soluble *starch synthase 1* (*SS1*) gene in potato protoplasts via ssODN‐templated gene editing, where most insertions were 10–30 nt in length, not the designed 6 nt, largely due to partial duplications of the 5′ homology arm at the Cas9 target site (González et al. 2025). Similarly, rearrangements were also observed in the *Nicotiana benthamiana NbPDS1* gene following Cas9/ssODN‐mediated editing. In these regenerated plants, the edited region was often characterized by the presence of 1–13 ssODN repeats, although some of these repeats were incomplete (Hsu et al. 2021). These results, which use ssODNs as DNA repair templates, indicate that in the cases mentioned above, most events involve sequence duplication regardless of whether Cas9 or Cas12a is used. Conversely, no sequence duplications were detected in *Arabidopsis thaliana* protoplasts during Cas9/ssODN‐mediated replacement of a proline with a serine in the *AtALS* gene (Jiang et al. 2021). However, only precise amino acid replacements in the *ALS* gene result in herbicide resistance, which would inhibit the recovery of additional mutations, including the sequence duplications observed above.

Besides ssODN‐derived sequence duplications, Cas12/ssODN‐mediated editing also triggered DNA rearrangements at the targeted alleles, resulting in allele absence from the amplicon libraries (labelled ∅) (Figure 6A), which may not be possible to observe by amplicon sequencing of a protoplast population (González et al. 2025), illustrating the importance of investigating the editing outcome at the plant level. A potential cause of amplification failure could be the presence of extended inverted repeats, as suggested by the inability to amplify the Bb29 mutated B1 allele by inverse PCR (Figure S10). In fact, the amplification of the WT *SteIF4E1_B* allele in the inverse PCR experiment with the B1‐specific reverse primer (Figure S10) could be in accord with a model in which polymerase B1‐extended reverse primer terminates prematurely due to structural constraints and reanneals to the WT *SteIF4E1_B* allele. Inverted sequences were detected in the B allele of lines Ba25 and Da118 (Figure S12), supporting this hypothesis.

Intriguingly, oligo‐mediated editing of eIF4E1 was confined to single alleles. Among 45 oligo‐edited potatoes (7.1% of regenerated plants), none had ssODN‐derived sequences in all four *eIF4E1* copies (Figure 6 and data not shown). Further, only one line out of 39 analysed by amplicon sequencing, Ba13, contained all the SNPs targeted for introduction via SSTR, representing 2.6% of edited lines. The presence of truncated oligo duplications in A38, Ba13, Ba44, Db100 and Db190 (Figure S12) suggests that ssODNs were not involved in homology‐mediated repair requiring extended DNA pairing, as expected for SSTR. Different scenarios could account for the observed imprecise DNA repair. ssODNs may have been degraded from both termini by exonucleases prior to incorporation at Cas12a DSBs via short sequence homologies of 1–2 nucleotides, consistent with MMEJ. In line Ba2 (Figure S12), sequence duplications were restricted to regions upstream of the Cas12a DSB. In these cases, ssODNs may have been used via synthesis‐dependent strand annealing at the 3′ end of the Cas12a cleavage site. The absence of 3′ SNPs in the edited sequences could be consistent with this DNA repair model. However, the presence of truncated sequence duplications at the 5′ end of the Cas12a cleavage site suggests that the newly synthesized strand may have been incorporated through short microhomologies at the 5′ end, rather than via extensive annealing involving the 5′ homology arms. Alternatively, extensive 3′ degradation of the ssODN may have resulted in loss of the intended 3′ and central SNPs, with the remaining ssODN fragments being integrated via MMEJ at both termini, thereby excluding SDSA.

Of note, in contrast to the roughly equal mutation frequencies across all four *SS1* alleles (González et al. 2025), we observed an allele‐specific preference for ssODN insertion at fully matching gRNA target sites. Analysis of the edited plants with unique genotypes (28 out of 39) (Table S3), to avoid possible duplication arising from protoplast regeneration, showed a clear preference for ssODN insertion in the B1 allele (19/28) compared to a single B allele (5/28). This suggests that factors such as chromatin structure, DNA methylation, or epigenetic properties may influence Cas cleavage efficacy and DNA repair outcomes (Assa et al. 2024; Cucuy et al. 2026; Přibylová et al. 2022; Weiss et al. 2022). Further studies are required to understand the underlying molecular mechanisms and to optimize this technology, especially for non‐dividing plant cells, such as the protoplasts used in this study, where NHEJ and MMEJ seem to dominate the DNA repair landscape.

### 3.2 PVY Resistance in the Dwarf Bb29 Potato Line Is eIF4E1‐Mediated

Despite the absence of intended *pvr2* ^*1*^‐mimic mutations in edited plants, we identified a unique line, Bb29, with genotype *AΔ12*, *BΔ6*, *B*, *B1pvr2^1^SD* (Figures 2, 5 and 6), which exhibited a strong resistance to three necrogenic PVY isolates, along with reduced systemic infection by PVY‐O and a dwarf habit (Figure 3 and Figure S6). Resistance was markedly greater than in *eIF4E1* knockout lines (Lucioli et al. 2022), which showed only partial reductions in PVY‐Pa36 accumulation and SHR response when challenged by PVY‐O. In contrast, Bb29 was rarely infected by multiple PVY‐NTN isolates (Figure 3A–C) and showed a strong amelioration of PVY‐O‐induced systemic HR (Figure 3E and Figure S8A), indicating a broader and more robust resistance. The absence of a systemic HR paralleled the marked reduction in PVY‐O accumulation in systemic tissues (Figure 3D and Figure S8B). Previous studies showed that eIF4E and eIF(iso)4E were required for systemic spread in different potyvirus–host interactions (Contreras‐Paredes et al. 2013; German‐Retana et al. 2008). In addition, a link between mutations in VPg and the systemic movement of some potyviruses has been reported (Rajamäki and Valkonen 2002; Schaad et al. 1997). Thus, the low propensity of PVY‐O to move long distances in Bb29 could be attributable to interference with the eIF4E/VPg network.

Several lines of evidence support eIF4E1‐mediated resistance in Bb29. First, the PVY‐Pa36 infections observed under high viral load were associated with increased viral accumulation over time (Figure 4A,B). Infections resulted from viral evolution, with amino acid changes in the viral VPg (Figure 4C), and Bb29 plants became fully susceptible to the resistance‐breaking PVY‐Pa36(K105E) isolate (Figure S9). This contrasts with *eIF4E1* knockouts, in which reduced viral accumulation was not associated with resistance‐breaking variants (Lucioli et al. 2022). These findings are consistent with previous reports that VPg mutations, particularly within amino acids 101–123, enable PVY to overcome *eIF4E*‐based resistance by restoring host compatibility or shifting isoform usage (Ayme et al. 2006, 2007; Charron et al. 2008; Janzac et al. 2014; Moury et al. 2004; Takakura et al. 2018). Second, ectopic expression of wild‐type *eIF4E1* restored both PVY susceptibility and growth in Bb29, confirming that resistance and dwarfism result from *eIF4E1* mutations (Figure 7A–D). Interestingly, ectopic wild‐type eIF4E1 expression reduced eIF4E2 expression (Figure 7D), highlighting regulatory networks that balance eIF4E protein levels. Previous research shows that knocking out or down one eIF4 family member can alter the expression of another, suggesting feedback mechanisms possibly at the post‐transcriptional level (Combe et al. 2005; Duprat et al. 2002; Gauffier et al. 2016; Lellis et al. 2019).

### 3.3 A Dominant‐Negative Resistance Mechanism Driven by the *SteIF4E1_AΔ12* Allele Underlies Bb29 Resistance

A key finding is that Bb29 resistance is consistent with a dominant‐negative mechanism. First, unlike classical recessive *S*‐gene resistance, which requires loss‐of‐function of all alleles, Bb29 retains a wild‐type *SteIF4E1* allele. cDNA sequencing confirmed expression of the *SteIF4E1_B* allele alongside alleles carrying 6‐ and 12‐nt deletions (Figure 5), indicating that resistance occurs despite the presence of a functional susceptibility factor.

Second, resistance in Bb29 is stronger and broader than in *SteIF4E1* knockouts (Lucioli et al. 2022), arguing that the resistance should not be the result of the inability of the eIF4E1 mutated protein(s) to interact with the VPg mimicking the absence of a susceptible factor, but rather its ability to interfere with the use of other members of the eIF4E family.

Third, the dwarf phenotype, absent in *SteIF4E1* knockout lines, supports interference with the broader eIF4E network. Consistently, combined disruption of *eIF4E* isoforms in tomato and tobacco leads to stunted growth, whereas single knockouts do not (Gauffier et al. 2016; Kumar et al. 2022). In addition, tobacco plants knocked down for *eIF4E* or for *eIFiso4E* displayed normal vegetative development, whereas antisense depletion of both *eIF4E* and *eIFiso4E* resulted in plants with a semi‐dwarf phenotype (Combe et al. 2005). Moreover, silencing of potato *eIF4E1* and *eIF4E2* resulted in stunted growth and reduced overall productivity (Miroshnichenko et al. 2025). Importantly, the PVY‐NTN VPg was shown to interact with potato eIF4E1 and eIF4E2 but not with eIF(iso)4E (Lebedeva et al. 2021), thus suggesting that in the dwarf Bb29, there is a potential interference with both eIF4E1 and eIF4E2 function that, however, does not imply an altered eIF4E2 expression (Figure 7E). The restoration of normal growth in Bb29 upon expression of wild‐type eIF4E1 further supports a dominant‐negative effect impacting translation factor balance.

Fourth, expression of the *SteIF4E1_AΔ12* allele in wild‐type Désirée recapitulates Bb29 resistance to PVY‐Pa36 and PVY‐O, even with occasional emergence of resistance‐breaking variants (Figure 8 and Figure S14). Previous studies have shown that overexpression of resistant *eIF4E* alleles can confer variable PVY resistance, the effect often depending on the genetic background (Arcibal et al. 2016; Cavatorta et al. 2011; Duan et al. 2012; Gutierrez Sanchez et al. 2020; Zhang et al. 2021), highlighting the influence of different native *eIF4E* allelic variants for viral interference. Here, the *SteIF4E1_AΔ12* was expressed in the native Désirée background. Given that the Bb29 *SteIF4E1_BΔ6* allele is identical to that of the PVY‐susceptible line Da114 (Figure 6 and Figure S11), and that *SteIF4E1_B1pvr2* ^*1*^ *SD*, if expressed, can encode a short truncated protein lacking domains essential for Cap, VPg and eIF4G binding (Miras et al. 2017; Lebedeva et al. 2024; Monzingo et al. 2007), *SteIF4E1_AΔ12* emerges as an important contributor to the dominant‐negative resistance in Bb29. Nonetheless, further work is needed to elucidate the underlying molecular mechanisms.

This is, to our knowledge, the first time that a CRISPR‐generated dominant‐negative resistance to a virus built on a pathogen‐susceptibility factor has been described. Our discovery may pave the way for new strategies in developing virus resistance against potyviruses.

## 4 Experimental Procedures

### 4.1 Plant Material and Potato Virus Y Strain

Nodal stem explants of cv. Désirée (NAK; plantenpaspoort zp‐d2/a6/a13 model 2643.454.408) were collected from sprouted potato tubers. Explants sterilization and in vitro culture were performed as described in Tavazza and Ancora (1986). PVY‐Pa36, PVY‐Pa21 and PVY‐O were kindly provided by Dr. Massimo Turina and Dr. Marina Ciuffo. PVY‐Hu was kindly provided by Dr. Jozsef Burgyan. PVY‐Pa21, PVY‐Pa36 and PVY‐Hu belong to the PVY‐NTN strain.

### 4.2 Cloning and Sequencing of eIF4E1 cDNAs of the Potato cv. Désirée and eIF4E cDNA of *A. thaliana*

Total RNA was extracted from potato cv. Désirée using Mirvana (Ambion) and reverse‐transcribed with SuperScript III RT (Invitrogen) using oligo(dT) as recommended by the supplier. cDNA was used as template for Pfx (Invitrogen) amplification using primers SteIF4E_Fw1 and SteIF4E_Rev696 (Table S4) (94°C 3 min, 30 cycles [94°C 15 s, 58°C 30 s, 68°C 1 min], 68°C 5 min) and the obtained fragment was cloned into the EcoRV site of pBluescript. Single clones were sequenced using M13 Fw primer, and three allelic forms were identified: A, B and B1, generating clones pBSK_St_eIF4E_A, pBSK_St_eIF4E_B and pBSK_St_eIF4E_B1, respectively. Primers AteIF4E_Fw1 and AteIF4E_Rev708 (Table S4) were used on *Arabidopsis* (Columbia) cDNA (kindly provided by Prof. Tavladoraki) for Pfx amplification (94°C 3 min, 35 cycles [94°C 15 s, 60°C 30 s, 68°C 1 min], 68°C 5 min) and the PCR product was cloned in the EcoRV site of pBluescript, obtaining pBSK_At_eIF4E.

### 4.3 eIF4E1 Mutagenesis

Mutagenesis of eIF4E sequences subcloned into pBSK was performed using the Q5 Site‐Directed Mutagenesis Kit from New England BioLabs, as recommended by the supplier, with primers listed in Table S4. Primers eIF4ESt2Pvr2_1Fw and eIF4ESt2Pvr2_1Re were used to generate mutant clones pBSK_St_eIF4E_A_pvr2^1^ and pBSK_St_eIF4e_B_pvr2^1^. In addition, primers eIF4ESt2Pvr2_1Fw and eIF4ESt1Pvr2_1Re were used for mutant clone pBSK_St_eIF4E_A_pvr2^1^_T. All clones were sequenced to verify mutations.

### 4.4 eIF4E1 Complementation Assays in *Saccharomyces cerevisiae*

Fragments HincII‐EcoRI from pBSK clones containing wild‐type and mutant potato sequences of eIF4E A and B alleles were cloned into the SmaI and EcoRI sites of p424GPD to obtain p424GPD_St_A, p424GPD_St_B, p424GPD_St_A_pvr2^1^, p424GPD_St_B_pvr2^1^, p424GPD_St_A_pvr2^1^T, p424GPD_Bb29_AΔ12 and p424GPD_Bb29_BΔ6. For the *A. thaliana* *eIF4E* sequence, the HindIII (blunt‐ended with T4 polymerase)‐EcoRI fragment of pBSK_At_eIF4e was cloned into the SmaI and EcoRI sites of p424GPD, obtaining p424GPD_At_eIF4E. All clones in p424GPD were sequenced with M13 Fw to verify the correctness of inserts and were used to transform *S. cerevisiae* JO55 as described in Agatep et al. (1998). JO55 lacks the endogenous functional *eIF4E* gene and depends on expression of a human eIF4E cDNA under a glucose‐repressible, galactose‐dependent promoter. Complementation assays were performed by comparing the growth of each transformed strain in medium containing 1% galactose and 1% raffinose (positive control medium) with the growth in medium containing 2% glucose. Briefly, three colonies for each transformed strain were grown in liquid dropout medium (Ura− Trp−) containing 1% galactose and 1% raffinose, then diluted to an OD_600_ of 0.05 (c. 5 × 10^4^ cells/mL). Ten microlitres of this cell suspension, together with 1:10 and 1:100 serial dilutions, were spotted on agar plates (Ura− Trp−) containing 1% galactose and 1% raffinose or 2% glucose as a carbon source. Negative and positive controls were the JO55 strain transformed with the p424GPD empty vector or p424GPD_At_eIF4E, respectively.

### 4.5 Protoplasts Isolation, Transfection and Regeneration of Edited Potato Plants

*Solanum tuberosum* ‘Désirée’ protoplasts were isolated, transfected, cultured and regenerated as described in Lucioli et al. (2022). Transfection was performed with donor ssDNA plus either Cpf1/gRNA ribonucleoprotein (RNP) complex or Cpf1/gRNA plasmid (see below). RNP complex was assembled by preincubating EnGen Lba Cas12a (Cpf1) (New England BioLabs) at a 1:3 M ratio with gRNA (Table S4) in 50 mM NaCl, 10 mM Tris–HCl, 10 mM MgCl_2_, 100 μg/mL bovine serum albumen (BSA) pH 7.9 at 37°C for 15 min. gRNA was purified before assembling RNP complex, using a modification of the enrichment procedure for small RNAs from total RNA samples of the mirVanaTM miRNA Isolation Kit (Life Technologies). Briefly, 300 μg (80 μL) of gRNA were mixed with 5 volumes of Lysis/Binding buffer, and 1/10 volume of miRNA Homogenate Additive was added to the mixture; after incubating 10 min on ice, ethanol was added to a final concentration of 55%, the mixture was purified by filtration through a Filter Cartridge, and the eluate was ethanol precipitated. Donor ssDNA (Table S4) obtained by Biolegio (Bpure protocol www.biolegio.com) was added to the RNP complex at room temperature, and 20 μL of the Cpf1/gRNA/ssDNA mix (5 μg/1.34 μg/22 μg) was used for every single protoplast's transfection (experiments A, Ba and Bb). In the case of protoplasts' transfection with plasmid DNA, 10 μg of pGem4Z_LbCpf1_St4E was mixed with either 22 or 35 μg of donor ssDNA (Da and Db transfection experiments, respectively), in 20 μL of 2.1 NEB buffer. Three independent transfections were conducted for each experiment.

### 4.6 Screening of Edited Potato Plants

Genomic DNA extracted from regenerants, as in Edwards et al. (1991), was used in a PCR with primers St2‐Pvr2^1^_Fw and St2‐Pvr2^1^_Rev (Table S4). Primer St2‐Pvr2^1^_Rev contains four nucleotides at its 3′ extremity that specifically recognize only edited plants (the sequence present in ssDNA used in Cpf1‐mediated editing). Amplified fragments were sequenced using St2‐Pvr2^1^_Fw as primer. Primers eIF4E_LEI_Fw20 and eIF4E_Con_Rev were used to selectively amplify wild‐type eIF4E sequences. Primers eIF4E_Con110_Fw and eIF4E_Con110_Rev were used to identify unwanted insertion/deletion in the edited region. PCR conditions were (94°C 2 min, 35 cycles [94°C 15 s, 60°C 30 s, 72°C 30 s], 72°C 5 min). All lines scoring editing‐positive were analysed twice, using two independent DNA extractions.

### 4.7 Cloning and Sequencing of eIF4E1 cDNAs From the Bb29 Edited Line and Inverse PCR

Total RNA was extracted from edited potato line Bb29 PVY‐resistant plant #552 according to Chomczynski and Sacchi (2006). Two micrograms were treated with Amplification‐grade DNase I (Invitrogen) and reverse‐transcribed with SuperScript III RT (Invitrogen) using oligo(dT) as recommended by the supplier. First‐strand cDNA was used as template for Pfx (Invitrogen) amplification using primers SteIF4E_Fw1 and SteIF4E_Rev696 (Table S4) (94°C 3 min, 35 cycles [94°C 15 s, 58°C 30 s, 68°C 45 s], 68°C 5 min) and the obtained fragment was cloned into the EcoRV site of pBluescript. Single clones were sequenced using M13Fw and M13Rev primers. Inverse PCR was performed on HincII‐digested Bb29 genomic DNA as in Green and Sambrook (2019). Primer sequences are reported in Table S4.

### 4.8 Generation of Targeted eIF4E1 Amplicon Libraries

To generate targeted amplicon sequencing libraries, DNA was extracted from wild‐type and gene‐edited plants. Subsequently, the eIF4E1‐specific genomic loci, spanning 320–324 bp around the CRISPR/Cas12a target site, were amplified using Q5 High‐Fidelity DNA Polymerase (New England BioLabs) and a pair of eIF4E1‐specific forward and reverse primers. These primers harboured 8‐nucleotide barcodes and partial Illumina adapter sequences (Table S4). Following amplification, the DNA was purified with AMPure XP beads (Beckman Coulter) according to the manufacturer's instructions and quantified using the Qubit dsDNA High‐Sensitivity Kit (Invitrogen). Equal amounts of DNA were then mixed to create a pooled sample which was sequenced on Genewiz's Amplicon‐EZ platform (https://web.genewiz.com/faqs/amplicon‐ez).

### 4.9 NGS Amplicon Sequencing Analysis—Mutation Events in Individual Samples and Alleles

Amplicon sequencing libraries were analysed and scored for quality by FastQC (RRID: SCR_014583), and individual samples were demultiplexed by their 5′ and 3′ barcodes using zgrep and trimmed using trimmomatic‐0.39 (RRID: SCR_011848). By an in‐house script with a combination of Bowtie2 (RRID: SCR_016368), individual samples were mapped to the alleles A, B and B1 using SNP positions to distinguish which allele each read belongs to; no mismatches were allowed at the SNP position using program Geneious Prime 2024‐to‐2025.1.1. Each subset of reads belonging to an individual sample and an allele was analysed with CRISPResso2 (Clement et al. 2019). Based on the ‘Alleles_frequency_table_around_sgRNA_*.txt’ output, mutation events were identified. To minimize background noise from libraries' cross‐contamination, a threshold for low‐abundant reads was applied, > 10 reads and > 15% occurrence of the event. The number of reads per individual sample and per allele is provided in Table S2. For nine samples, biological replicates were generated (genomic DNA isolated from sister plants), and the analysis yielded the same conclusions (data not shown). The presence of 5′, 3′ and the whole length of the wanted sequence was analysed in individual samples and alleles. The threshold for low‐abundant reads was not applied to avoid filtering reads with mapping problems caused by the extensive presence of INDELSUB.

### 4.10 Virus Resistance Analysis

In vitro‐multiplicated *S. tuberosum* plants were acclimated to soil and PVY‐inoculated as described in Lucioli et al. (2022). Between 21 and 30 dpi, unless otherwise stated, upper leaves were sampled and assessed for PVY accumulation by DAS‐ELISA using a commercial BIOREBA kit (Art‐No: 110575). Absorbance was measured with the Multiskan FC (Thermo Fisher Scientific) microplate reader using SkanIt software 3.1.

### 4.11 Sequence Analysis of the PVY‐Pa36 Resistance‐Breaking Isolates

Total RNA was extracted from PVY‐inoculated potato plants according to the method of Chomczynski and Sacchi (2006). Reverse transcription, PCR amplification, and sequencing of the VPg region were performed as described in Lucioli et al. (2022).

### 4.12 Transgenic Plants

Fragments HindIII‐EcoRI from pBSK_St_eIF4E_A, pBSK_St_eIF4E_B and pBSK_Bb29_AΔ12 containing the coding sequence for eIF4E A and B alleles of wild‐type plants, or the mutated eIF4E_AΔ12 from the Bb29 edited line, were cloned into the corresponding sites of pJIT60, generating pJIT60_St_eIF4E_A, pJIT60_St_eIF4E_B and pTIT60_Bb29_AΔ12. Fragments KpnI‐XhoI from these three plasmids were cloned into the corresponding sites on pBin20, obtaining pBin20_St_eIF4E_A, pBin20_St_eIF4E_B and pBin20_Bb29_AΔ12, which were sequenced (see Table S4 for primers) and introduced into *A. tumefaciens* LBA4404.

Plants were regenerated on selective medium containing 50 μg/mL kanamycin; genomic DNA was extracted as in Edwards et al. (1991) and PCR‐amplified to confirm transgene integration. Primers SteIF4E_Fw1 and SteiF4E_Rev696 (Table S4) were used to confirm integration of eIF4E A and B sequences, primers SteIF4E_Fw20 and Bb29_RevΔ12 (Table S4) to confirm integration of mutated Bb29_AΔ12, and primers Univ and Rev. to confirm integration of pBin20 empty vector sequences.

### 4.13 Protein Extraction and Western Blot Analysis

Yeast proteins were extracted as described in Zhang et al. (2011). Total proteins were extracted from potato leaves and stems by boiling in 6 volumes of Laemmli buffer. For pull‐down experiments with the cap analogue g‐Aminophenil‐m7GTP (C10‐spacer)‐agarose (Jena Bioscience), total soluble proteins were extracted from 50–70 mg leaves by grinding in liquid nitrogen and homogenizing in 300 μL binding buffer (Zafirov et al. 2023); binding and washing were performed as in Zafirov et al. (2023).

After SDS‐PAGE on a 12% gel, proteins were transferred to a PVDF membrane (semidry blotting) and incubated with rabbit anti‐tobacco eIF4E1‐like antibodies (#991) (Combe et al. 2005).

## Author Contributions

**Adéla Přibylová:** writing – review and editing, investigation, validation, software. **Raffaela Tavazza:** investigation, validation, writing – review and editing. **Alessandra Lucioli:** investigation, data curation, validation. **Vincenza Ilardi:** writing – review and editing, investigation. **Attila Molnar:** conceptualization, investigation, validation, writing – review and editing, writing – original draft. **Mario Tavazza:** investigation, validation, conceptualization, writing – original draft, writing – review and editing, supervision.

## Conflicts of Interest

The authors declare no conflicts of interest.

## Supporting information


**Figure S1:** Nucleotide alignment of *SteIF4E1* alleles of potato cv. Désirée. *SteIF4E1* alleles were deduced from 31 SteIF4E1 cDNA sequences. Red nucleotides, SNPs differing from the *SteIF4E1_A* allele sequence. Arrows, forward and reverse primers used for *SteIF4E1* cDNA amplification.

**Figure S2:** Amino acid alignment of proteins encoded by *A*, *B* and *B1 eIF4E1* alleles of potato cv. Désirée. Red letters, amino acids differing from the *SteIF4E1_A* allele. The black box identifies the amino acid region (70–82) subjected to mutagenesis.

**Figure S3:** Yeast complementation analyses of *SteIF4E1_Apvr2* ^*1T*^ allele. (A) Amino acid sequences of the region I (Poulicard et al. 2016) of pepper and potato eIF4E1 proteins. *pvr2* ^*+*^ and *pvr2* ^*1*^ PVY‐susceptible and resistant pepper *eIF4E* allele, respectively; *SteIF4E1_A*, wild‐type potato *A* allele; *SteIF4E1_Apvr2* ^*1T*^, potato *A* allele mimicking *pvr2* ^*1*^, mutated amino acids are marked in red. (B) The yeast strain JO55 was transformed with either an empty p424GPD plasmid (negative control) or p424GPD constructs expressing *SteIF4E1_A* (positive control) and the St*eIF4E1_Apvr2* ^*1T*^ allele. Dilutions were spotted on galactose/raffinose (Gal/Raf) synthetic dropout medium lacking uracil and tryptophan and on a selective nutrient dropout medium containing glucose and lacking uracil and tryptophan.

**Figure S4:** Alignment of potato Désirée *SteIF4E* sequences in the region encompassing *SteIF4E1* Cas12a‐mediated editing. Red box, Cas12a PAM site. (A) alignment of *SteIF4E1* and *SteIF4E2* sequences; (B) manual alignment of *SteIF4E1* and *SteIF(iso)4E* sequences. *SteIF4E2* and *SteIF(iso)4E* sequences were inferred from Sevestre et al. (2020).

**Figure S5:** PCR analysis of Cas12a‐edited potato plants. (A) Nucleotide sequence of the first exon of the *SteIF4E1_B* allele. In italics, the sequence region used as ssDNA for Cas12a‐mediated editing. Nucleotides differing from the *SteIF4E1_B* are marked in red. Primer sequences used for the screening are underlined. Boxed sequence indicates Cas12a PAM site. (B) PCR analysis of edited potato plants. A, Ba, Bb, Da and Db indicate independent protoplasts transfection experiments. For A, Ba and Bb, transfections were performed using Cas12a‐gRNA ribonucleotide complex plus ssODN, whereas for Da and Db pGem4Z_LbCpf1_St4E plasmid and ssODN were used. Control un‐edited plants are marked in red. M, GeneRuler 100 bp DNA ladder; the size of the upper visible GeneRuler DNA band is 500 bp.

**Figure S6:** Screening for PVY resistance of Cas12a‐edited potato plants. Wild‐type and edited potato lines were challenged with PVY‐Pa36, and virus accumulation was assessed between 21 and 30 days post‐inoculation by double‐antibody sandwich enzyme‐linked immunosorbent assay. A pool of extracts from the wild‐type PVY‐Pa36‐infected plants was used in a two‐fold dilution series to assess virus accumulation.

**Figure S7:** Hypersensitive response elicited by PVY‐O on the inoculated leaves of wild‐type and Bb29 Désirée plants. Two consecutive leaves of each plant were inoculated with the virus, L and U for lower and upper, respectively. The images were captured at the time reported on the left; dpi, days post‐inoculation.

**Figure S8:** PVY‐O‐induced systemic necrosis in wild‐type (WT) Désirée potatoes is markedly reduced in Bb29 plants. (A) Comparison of WT and Bb29 plants at 14 dpi. Extended leaf necrosis develops in WT plants, while occasionally single lesions may occur in some Bb29 plants. Ctrl, uninoculated control WT plant. (B) Virus accumulation in systemic plant tissues of WT and Bb29 plants at 14 days post‐inoculation (dpi). A pool of extracts from the four WT plants was used in a two‐fold dilution series to assess virus accumulation.

**Figure S9:** Resistance analysis of Bb29 plants challenged with the resistance breaking PVY‐Pa36(K105E) isolate. Virus accumulation was assed at 21 days post‐inoculation by double‐antibody sandwish‐ELISA. Centre lines show the medians; box limits indicate the 25th and 75th percentiles, as determined by R. The numbers above the boxes indicate the number of plants analysed.

**Figure S10:** Inverse PCR for sequencing Bb29 *eIF4E1_B1* mutated allele. (A) Schematic representation of the Bb29 genomic region surrounding exon 1 of the B1 allele. (B) Schematic representation as in (A), after HincII digestion and intramolecular ligation. Arrows represent primers used for PCR amplification and DNA sequencing. In the sequence below, red lowercase letters represent mutated nucleotides mimicking *pvr2* ^*1*^, and light blue, italicized duplicated sequences. (C) Sequencing results of the PCR fragment obtained using primers indicated.

**Figure S11:** Yeast complementation analyses of *SteIF4E1_A* and *SteIF4E1_B* mutated alleles of Bb29 potato plants. (A) Amino acid sequences of the region I (Poulicard et al. 2016) of SteIF4E1 proteins. SteIF4E1_A and SteIF4E1_B wild‐type proteins; SteIF4E1_AΔ12 and SteIF4E1_BΔ6 proteins derived from *SteIF4E1_A* and *B Bb29* mutated alleles. Deleted amino acids are represented with en‐dashes. (B) The yeast strain JO55 was transformed with either an empty p424GPD plasmid (negative control) or with p424GPD constructs expressing *SteIF4E1_A* and *SteIF4E1_B* (positive controls) and Bb29 mutated *SteIF4E1_AΔ12* and *SteIF4E1_BΔ6* alleles. Dilutions were spotted on galactose/raffinose (Gal/Raf) medium and on a selective medium containing glucose. Only yeast functionally complemented by the ectopic expression of an eIF4E protein can grow on the selective medium. (C) Western blot of protein extracts from yeast strain JO55 transformed with (1) p424GPD_*SteIF4E1_A*; (2 and 4) p424GPD_*SteIF4E1_B*; (3) p424GPD empty vector; (5) p424GPD_*SteIF4E1_AΔ12*; (6) p424GPD_*SteIF4E1_BΔ6*. Yeast was grown on galactose and raffinose as carbon sources, except for yeast in line 4, which was grown on glucose. The lower panel shows Coomassie staining of a replica gel to assess protein loading.

**Figure S12:** Sequence analysis of *eIF4E1* alleles containing pvr2^1^SD mutations. For each edited line, the mutated sequence is aligned with the theoretical mutated sequence. Red letters: mutated nucleotides in the ssODN. Duplicated sequences are underlined and identified as x1 and x2 (with × spanning from A to M) and the x2 sequence is in light blue. When the duplicated sequence is in the inverted orientation, the repeated inverted sequence is in light blue italic. Boxed sequences: Cas12a PAM site. Bold letters: stop codon.

**Figure S13:** Height analysis of uninoculated Bb29 plants transformed with *SteIF4E1_A* allele (A lines), *SteIF4E1_B* allele (B lines) or an empty vector (V lines) as a control. (A). The label DN stands for the wild‐type Désirée. Plants were photographed at time zero when the other plants in the pair were inoculated with PVY‐Pa36. (B) Plants at 21 days after time zero.

**Figure S14:** PVY‐Pa36 resistance analyses of wild‐type Désirée plants transformed with the Bb29 mutated *SteIF4E1_AΔ12* allele. Virus accumulation was evaluated at 30 days post‐inoculation by double‐antibody sandwich‐ELISA. D12‐4, D12‐17, D12‐42, D12‐59, D12‐98 transgenic lines for the *SteIF4E1_AΔ12* allele. P7 or P12 transgenic lines for the empty vector. Control, non‐inoculated P7 or P12 plant. The sap from P7 or P12 plants was mixed and used to generate the standard curve.

**Table S1:** mpp70305‐sup‐0015‐TableS1.docx. *SteIF4E1_pvr2* ^*1*^ potato editing.

**Table S2:** Extended amplicon sequencing data.

**Table S3:** Twenty‐eight potato edited plants have an unique genotype.

**Table S4:** Oligonucleotides used.

## Data Availability

The data that support the findings of this study are available from the corresponding author upon reasonable request.
