# Supplementary material for: Imprecise Cas12a/ssODN‐Mediated Editing of eIF4E1 Confers Dominant‐Negative Resistance to Potato Virus Y in Solanum tuberosum
Source: Mol Plant Pathol. 2026 Jun 30;27(7):e70305. doi: 10.1111/mpp.70305 (PMC13315812; doi:10.1111/mpp.70305)
Supplement: Supplementary file 1 — Figure S1: Nucleotide alignment of SteIF4E1 alleles of potato cv. Désirée. SteIF4E1 alleles were deduced from 31 SteIF4E1 cDNA sequences. Red nucleotides, SNPs differing from the SteIF4E1_A allele sequence. Arrows, forward and reverse primers used for SteIF4E1 cDNA amplification. [file MPP-27-e70305-s012.pdf]

```
SteIF4E1_A      ATGGCAGCAGCTGAAATGGAGAGAACGACGTCGTTTGATGCAGCTGAGAAGTTGAAGGCC
SteIF4E1_B      ATGGCAGTAGCTGAAATGGAGAGAACGACGTCGTTTGATGCAGCTGAGAAGTTGAAGGCC
SteIF4E1_B1     ATGGCAGCAGCTGAAATGGAGAGAACGACGTCGTTTGATGCAGCTGAGAAGTTGAAGGCC
                ******* ****************************************************

SteIF4E1_A      GCCGATGCAGGAGGAGGAGAGGTAGACGATGAACTTGAAGAAGGTGAAATTGTTGAAGAA
SteIF4E1_B      GCCGATGGAGGAGGAGGGGAGGTAGACGATGAACTTGAAGAAGGTGAAATTGTTGAAGAA
SteIF4E1_B1     GCCGATGCAGGAGGAGGAGAGGTAGACGATGAACTTGAAGAAGGTGAAATTGTTGAAGAA
                ******* ********* ******************************************

SteIF4E1_A      TCAAATGATACGGCGTCGTATTTAGGGAAAGAAATCACAGTGAAACATCCATTGGAGCAT
SteIF4E1_B      TCAAATGATATGGCGTCGTATTTAGGGAAAGAAATCACAGTGAAGCATCCATTGGAGCAT
SteIF4E1_B1     TCAAATGATACGGCGTCGTATTTAGGGAAAGAAATCACAGTGAAGCATCCATTGGAGCAT
                ********** ********************************* ***************

SteIF4E1_A      TCATGGACTTTTTGGTTTGATAGCCCTATTGCTAAATCTCGACAAACTGCTTGGGGAAGC
SteIF4E1_B      TCATGGACTTTTTGGTTTGATAGCCCTATTGCTAAATCTCGACAAACTGCTTGGGGAAGC
SteIF4E1_B1     TCATGGACTTTTTGGTTTGATAGCCCTATTGCTAAATCTCGACAAACTGCTTGGGGAAGC
                ************************************************************

SteIF4E1_A      TCACTTCGAAATGTCTACACTTTCTCCACTGTTGAAGATTTTTGGGGTGCTTACAATAAT
SteIF4E1_B      TCACTTCGAAATGTCTACACTTTCTCCACTGTTGAAGATTTTTGGGGTGCTTACAATAAT
SteIF4E1_B1     TCCCTTCGAAATGTCTACACTTTCTCCACTGTTGAAGATTTTTGGGGTGCTTACAATAAT
                ** *********************************************************

SteIF4E1_A      ATCCATCACCCAAGCAAGTTGGTTATGGGAGCAGACTTTCATTGTTTTAAGCATAAAATT
SteIF4E1_B      ATCCATCACCCAAGCAAGTTGGTTATGGGAGCAGACTTTCATTGTTTTAAGCATAGAATT
SteIF4E1_B1     ATCCATCACCCAAGCAAGTTGGTTATGGGAGCAGACTTTCATTGTTTTAAGCATAAAATT
                ******************************************************* ****

SteIF4E1_A      GAGCCAAAGTGGGAAGATCCTGTATGTGCCAATGGAGGGACGTGGAAAATGAATTTTTTG
SteIF4E1_B      GAGCCAAAGTGGGAAGATCCTGTATGTGCCAATGGAGGGACGTGGAAAATGAGTTTTTCG
SteIF4E1_B1     GAGCCAAAGTGGGAAGATCCTGTATGTACCAATGGAGGGACGTGGAAAATGAGTTTTTCG
                *************************** ************************ ***** *

SteIF4E1_A      AAGGGTAAATCTGATACCAGCTGGCTATATACGCTGCTGGCAATGATTGGACATCAATTC
SteIF4E1_B      AAGGGTAAATCTGATACCAGCTGGCTATATACGCTGCTGGCAATGATTGGACATCAATTC
SteIF4E1_B1     AAGGGTAAATCTGATACCAGCTGGCTATATACGCTGCTGGCTATGATTGGACATCAATTT
                ***************************************** *****************

SteIF4E1_A      GATCACGGAGATGAAATTTGTGGAGCAGTCGTTAGTGTCCGGTCTAAGGGAGAAAAAATA
SteIF4E1_B      GATCATGGAGATGAAATTTGTGGAGCAGTCGTTAGTGTCCGGGCTAAGGGAGAAAAAATA
SteIF4E1_B1     GATCATGGAGATGAAATTTGTGGAGCAGTCGTTAGTGTCCGGGCTAAGGGAGAAAAAATA
                ***** ************************************ *****************

SteIF4E1_A      GCTTTGTGGACCAAGAATGCTGCAAATGAAACAGCTCAGGTTAGCATTGGTAAGCAATGG
SteIF4E1_B      GCTTTGTGGACCAAGAATGCTGCAAATGAAACAGCTCAGGTTAGCATTGGTAAGCAATGG
SteIF4E1_B1     GCTTTGTGGACCAAGAATGCTGCAAATGAAACAGCTCAGGTTAGCATTGGTAAGCAATGG
                ************************************************************

SteIF4E1_A      AAGCAGTTTCTAGATTACAGCGATTCGGTTGGCTTCATATTTCACGATGATGCAAAGAGG
SteIF4E1_B      AAGCAGTTTCTAGATTATAGCGATTCGGTTGGCTTCATATTTCATGACGATGCAAAGAGG
SteIF4E1_B1     AAGCAGTTTCTAGATCATAGCGATTCGGTTGGCTTCATATTTCATGACGATGCAAAGAGG
                *************** * ************************** ** ************

SteIF4E1_A      CTCGACAGAAGTGCCAAGAATCGTTACACCGTATGA
SteIF4E1_B      CTCGACAGAAATGCCAAGAATCGTTACACCGTATGA
SteIF4E1_B1     CTCGACAGAAATGCCAAGAATCGTTACACCGTATGA
                ********** *************************
```

Figure S1. Nucleotide alignment of *SteIF4E1* alleles of potato cv Désirée. *SteIF4E1* alleles were deduced from 31 SteIF4E1 cDNA sequences. Red nucleotides, SNPs differing from the *SteIF4E1_A* allele sequence. Arrows, forward and reverse primers used for *SteIF4E1* cDNA amplification.
