# Supplementary material for: Imprecise Cas12a/ssODN‐Mediated Editing of eIF4E1 Confers Dominant‐Negative Resistance to Potato Virus Y in Solanum tuberosum
Source: Mol Plant Pathol. 2026 Jun 30;27(7):e70305. doi: 10.1111/mpp.70305 (PMC13315812; doi:10.1111/mpp.70305)
Supplement: Supplementary file 2 — Figure S2: Amino acid alignment of proteins encoded by A, B and B1 eIF4E1 alleles of potato cv. Désirée. Red letters, amino acids differing from the SteIF4E1_A allele. The black box identifies the amino acid region (70–82) subjected to mutagenesis. [file MPP-27-e70305-s016.pdf]

```
eIF4E1_B    MAVAEMERTTSFDAAEKLKAADGGGGEVDDELEEGEIVEESNDMASYLGKEITVKHPLEH 60
eIF4E1_A    MAAAEMERTTSFDAAEKLKAADAGGGEVDDELEEGEIVEESNDTASYLGKEITVKHPLEH 60
eIF4E1_B1   MAAAEMERTTSFDAAEKLKAADAGGGEVDDELEEGEIVEESNDTASYLGKEITVKHPLEH 60
            **.**********************.***************** ****************

eIF4E1_B    SWTFWFDSPIAKSRQTAWGSSLRNVYTFSTVEDFWGAYNNIHHPSKLVMGADFHCFKHRI 120
eIF4E1_A    SWTFWFDSPIAKSRQTAWGSSLRNVYTFSTVEDFWGAYNNIHHPSKLVMGADFHCFKHKI 120
eIF4E1_B1   SWTFWFDSPIAKSRQTAWGSSLRNVYTFSTVEDFWGAYNNIHHPSKLVMGADFHCFKHKI 120
            **********************************************************:*

eIF4E1_B    EPKWEDPVCANGGTWKMSFSKGKSDTSWLYTLLAMIGHQFDHGDEICGAVVSVRAKGEKI 180
eIF4E1_A    EPKWEDPVCANGGTWKMNFLKGKSDTSWLYTLLAMIGHQFDHGDEICGAVVSVRSKGEKI 180
eIF4E1_B1   EPKWEDPVCTNGGTWKMSFSKGKSDTSWLYTLLAMIGHQFDHGDEICGAVVSVRAKGEKI 180
            *********:*******.* **********************************:*****

eIF4E1_B    ALWTKNAANETAQVSIGKQWKQFLDYSDSVGFIFHDDAKRLDRNAKNRYTV 231
eIF4E1_A    ALWTKNAANETAQVSIGKQWKQFLDYSDSVGFIFHDDAKRLDRSAKNRYTV 231
eIF4E1_B1   ALWTKNAANETAQVSIGKQWKQFLDHSDSVGFIFHDDAKRLDRNAKNRYTV 231
            *************************:*****************.*******
```

Figure S2. Amino acid alignment of proteins encoded by *A*, *B*, and *B1 eIF4E1* alleles of potato cv. Désirée.
Red letters, amino acids differing from the *SteIF4E1_A* allele. The black box identifies the amino acid region (70-82) subjected to mutagenesis.
