# Supplementary material for: Imprecise Cas12a/ssODN‐Mediated Editing of eIF4E1 Confers Dominant‐Negative Resistance to Potato Virus Y in Solanum tuberosum
Source: Mol Plant Pathol. 2026 Jun 30;27(7):e70305. doi: 10.1111/mpp.70305 (PMC13315812; doi:10.1111/mpp.70305)
Supplement: Supplementary file 3 — Figure S3: Yeast complementation analyses of SteIF4E1_Apvr2 1T allele. (A) Amino acid sequences of the region I (Poulicard et al. 2016) of pepper and potato eIF4E1 proteins. pvr2 + and pvr2 1 PVY‐susceptible and resistant pepper eIF4E allele, respectively; SteIF4E1_A, wild‐type potato A allele; SteIF4E1_Apvr2 1T , potato A allele mimicking pvr2 1 , mutated amino acids are marked in red. (B) The yeast strain JO55 was transformed with either an empty p424GPD plasmid (negative control) or p424GPD constructs expressing SteIF4E1_A (positive control) and the SteIF4E1_Apvr2 1T allele. Dilutions were spotted on galactose/raffinose (Gal/Raf) synthetic dropout medium lacking uracil and tryptophan and on a selective nutrient dropout medium containing glucose and lacking uracil and tryptophan. [file MPP-27-e70305-s014.pdf]

A

	67												79
Pvr2+	V	A	K	S	K	Q	A	A	W	G	S	S	L
Pvr2[1]	E	A	K	S	K	Q	A	A	W	G	S	S	R

	70												82
SteIF4E1_A	I	A	K	S	R	Q	T	A	W	G	S	S	L
SteIF4E1_Apvr2[1T]	E	A	K	S	R	Q	T	A	W	G	S	S	R

B

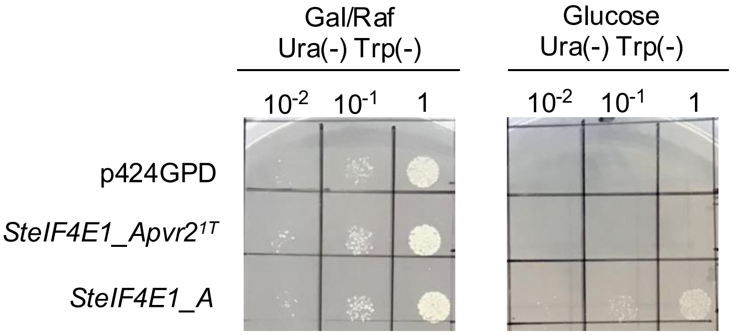

Figure S3. Yeast complementation analyses of *SteIF4E1_Apvr2[1T]* allele

(A) Amino acid sequences of the region I (Poulicard et al., 2016) of pepper and potato eIF4E1 proteins. *pvr2*+ and *pvr2[1]* PVY-susceptible and resistant pepper *eIF4E* allele, respectively; *SteIF4E1_A*, wild-type potato *A* allele; *SteIF4E1_Apvr2[1T]*, potato *A* allele mimicking *pvr2[1]*, mutated amino acids are marked in red.
(B) The yeast strain JO55 was transformed with either an empty p424GPD plasmid (negative control) or p424GPD constructs expressing *SteIF4E1_A* (positive control) and the Ste*IF4E1_Apvr2[1T]* allele. Dilutions were spotted on galactose/raffinose (Gal/Raf) synthetic drop-out medium lacking uracil and tryptophan and on a selective nutrient drop-out media containing glucose and lacking uracil and tryptophan.
