# Supplementary material for: Imprecise Cas12a/ssODN‐Mediated Editing of eIF4E1 Confers Dominant‐Negative Resistance to Potato Virus Y in Solanum tuberosum
Source: Mol Plant Pathol. 2026 Jun 30;27(7):e70305. doi: 10.1111/mpp.70305 (PMC13315812; doi:10.1111/mpp.70305)
Supplement: Supplementary file 4 — Figure S4: Alignment of potato Désirée SteIF4E sequences in the region encompassing SteIF4E1 Cas12a‐mediated editing. Red box, Cas12a PAM site. (A) alignment of SteIF4E1 and SteIF4E2 sequences; (B) manual alignment of SteIF4E1 and SteIF(iso)4E sequences. SteIF4E2 and SteIF(iso)4E sequences were inferred from Sevestre et al. (2020). [file MPP-27-e70305-s010.pdf]

A

```
SteIF4E1     ATCCATTGGAGCATTCATGGACTTTTTGGTTTGATAGCCCTATTGCTAAATCTCGACAAA
             ::::: : :: ::::: ::::: :::::::: :::: ::::   :  :::::   ::::
SteIF4E2     ATCCACTAGAACATTCTTGGACATTTTGGTTCGATAACCCTTCAGGGAAATCGAAACAAG
```

B

```
SteIF4E1        ATCCATTGGAGCATTCATGGACTTTTTGGTTTGATAGCCCTATTGCTAAATCTCGACAAA
                ::    : :::      ::::: :: ::::: :::: ::    :           :
SteIF(iso)4E    ATAAGCTAGAGAGGAAGTGGACGTTCTGGTTCGATAACCAGTCTAAACCGAAACAAGGCG
```

Figure S4. Alignment of potato Desirèe *SteIF4E* sequences in the region encompassing *SteIF4E1* Cas12a-mediated editing. Red box, Cas12a PAM site. (A) alignment of *SteIF4E1* and *SteIF4E2* sequences; (B) manual alignment of *SteIF4E1* and *SteIF(iso)4E* sequences. *SteIF4E2* and *SteIF(iso)4E* sequences were inferred from Sevestre et al. (2020).
