# Supplementary material for: Imprecise Cas12a/ssODN‐Mediated Editing of eIF4E1 Confers Dominant‐Negative Resistance to Potato Virus Y in Solanum tuberosum
Source: Mol Plant Pathol. 2026 Jun 30;27(7):e70305. doi: 10.1111/mpp.70305 (PMC13315812; doi:10.1111/mpp.70305)
Supplement: Supplementary file 5 — Figure S5: PCR analysis of Cas12a‐edited potato plants. (A) Nucleotide sequence of the first exon of the SteIF4E1_B allele. In italics, the sequence region used as ssDNA for Cas12a‐mediated editing. Nucleotides differing from the SteIF4E1_B are marked in red. Primer sequences used for the screening are underlined. Boxed sequence indicates Cas12a PAM site. (B) PCR analysis of edited potato plants. A, Ba, Bb, Da and Db indicate independent protoplasts transfection experiments. For A, Ba and Bb, transfections were performed using Cas12a‐gRNA ribonucleotide complex plus ssODN, whereas for Da and Db pGem4Z_LbCpf1_St4E plasmid and ssODN were used. Control un‐edited plants are marked in red. M, GeneRuler 100 bp DNA ladder; the size of the upper visible GeneRuler DNA band is 500 bp. [file MPP-27-e70305-s008.pdf]

A

ATGGCAGTAGCTGAAATGGAGAGAACGACGTCGTTTGATGCAGCTGAGAAGTTGAAGGCC 60

GCCGATGGAGGAGGAGGGGAGGTAGACGATGAACTTGAAGAAGGTGAAATTGTTGAAGAA 120

eIF4E_Con110_Fw

TCAAATGATATGGCGTCGTATTTAGGGAAAGAAATCACAGTGAAGCA*TCCATTGGAGCAT* 180

TCATGGACTTTTTGGTTTGATAGCCCAGAGGCTAAATCTCGACAAGCTGCTTGGGGAAGC 240

eIF4E_Con110_Rev

TCAAGACGAAATGTCTACACTTTCTCCACTGTTGAAGATTTTTGGG 286

B

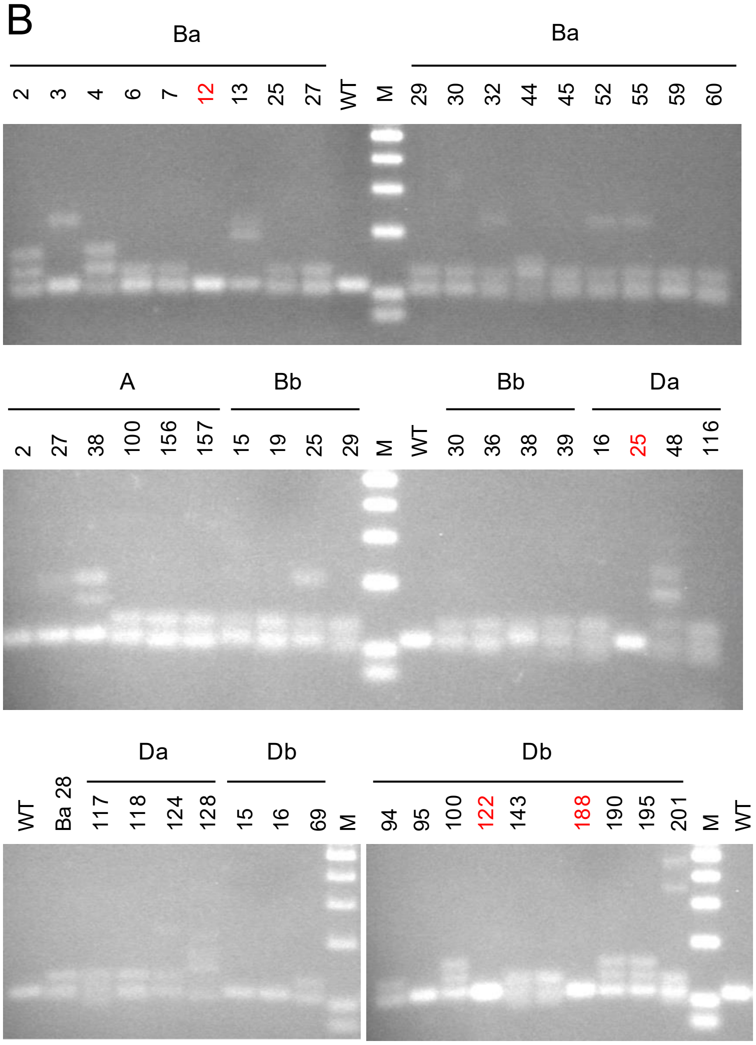

Figure S5. PCR analysis of Cas12a-edited potato plants. (A) Nucleotide sequence of the first exon of the *StelF4E1_B* allele. In italics, the sequence region used as ssDNA for Cas12a-mediated editing. Nucleotides differing from the *StelF4E1_B* are marked in red. Primer sequences used for the screening are underlined. Boxed sequence indicates Cas12a PAM site. (B) PCR analysis of edited potato plants. A, Ba, Bb, Da, and Db indicate independent protoplasts transfection experiments. For A, Ba, and Bb, transfections were performed using Cas12a-gRNA ribonucleotide complex plus ssODN, whereas for Da and Db pGem4Z_LbCpf1_St4E plasmid and ssODN were used. Control un-edited plants are marked in red. M, GeneRuler 100bp DNA ladder; the size of the upper visible GeneRuler DNA band is 500bp.
