# Supplementary material for: Imprecise Cas12a/ssODN‐Mediated Editing of eIF4E1 Confers Dominant‐Negative Resistance to Potato Virus Y in Solanum tuberosum
Source: Mol Plant Pathol. 2026 Jun 30;27(7):e70305. doi: 10.1111/mpp.70305 (PMC13315812; doi:10.1111/mpp.70305)
Supplement: Supplementary file 6 — Figure S6: Screening for PVY resistance of Cas12a‐edited potato plants. Wild‐type and edited potato lines were challenged with PVY‐Pa36, and virus accumulation was assessed between 21 and 30 days post‐inoculation by double‐antibody sandwich enzyme‐linked immunosorbent assay. A pool of extracts from the wild‐type PVY‐Pa36‐infected plants was used in a two‐fold dilution series to assess virus accumulation. [file MPP-27-e70305-s007.pdf]

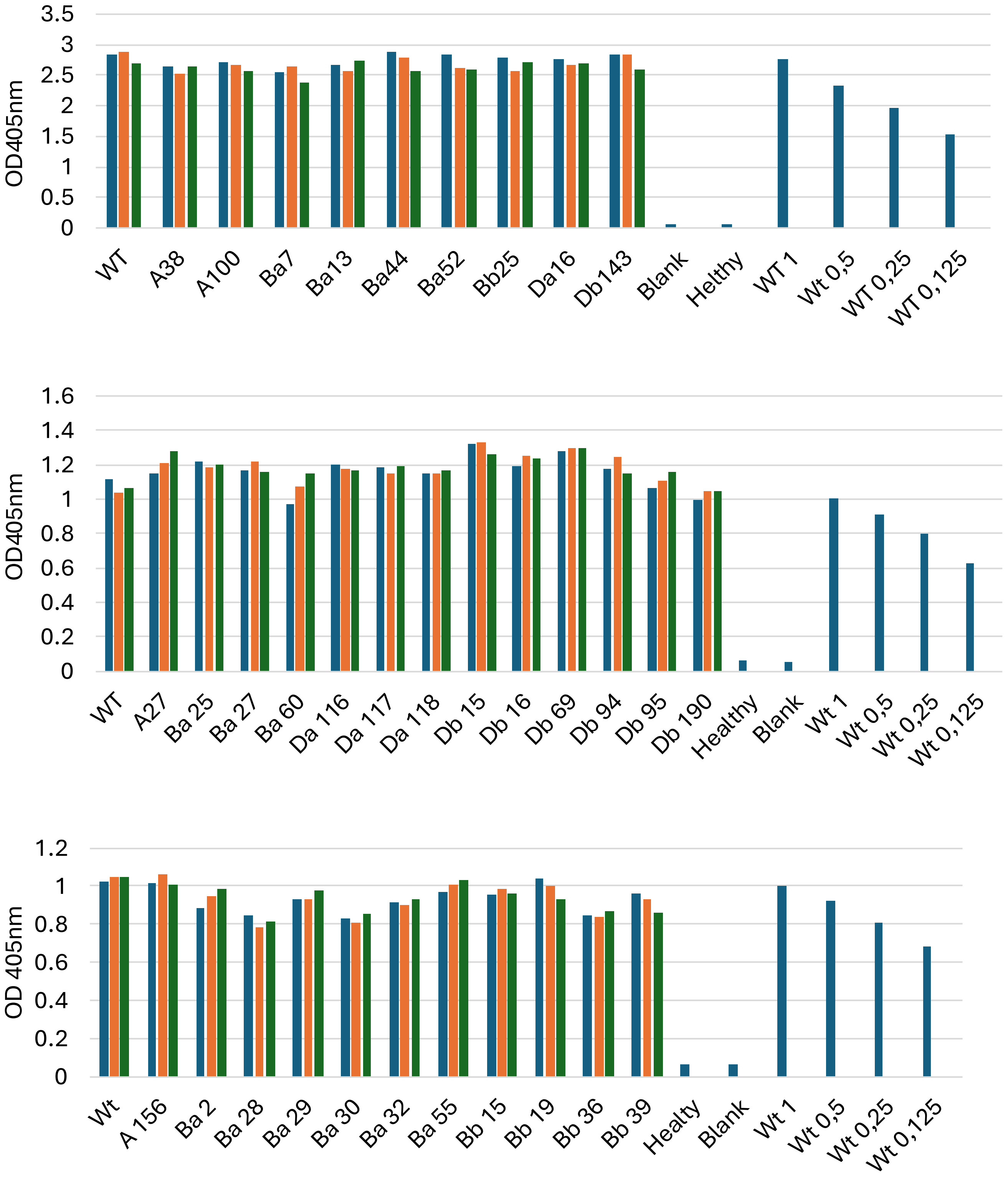
OD405nm
3.5
3
2.5
2
1.5
1
0.5
0
WT
A38
A100
Ba7
Ba13
Ba44
Ba52
Bb25
Da16
Db143
Blank
Helthy
WT 1
Wt 0,5
WT 0,25
WT 0,125
OD405nm
1.6
1.4
1.2
1
0.8
0.6
0.4
0.2
0
WT
A27
Ba 25
Ba 27
Ba 60
Da 116
Da 117
Da 118
Db 15
Db 16
Db 69
Db 94
Db 95
Db 190
Healthy
Blank
Wt 1
Wt 0,5
Wt 0,25
Wt 0,125
OD 405nm
1.2
1
0.8
0.6
0.4
0.2
0
Wt
A 156
Ba 2
Ba 28
Ba 29
Ba 30
Ba 32
Ba 55
Bb 15
Bb 19
Bb 36
Bb 39
Healty
Blank
Wt 1
Wt 0,5
Wt 0,25
Wt 0,125

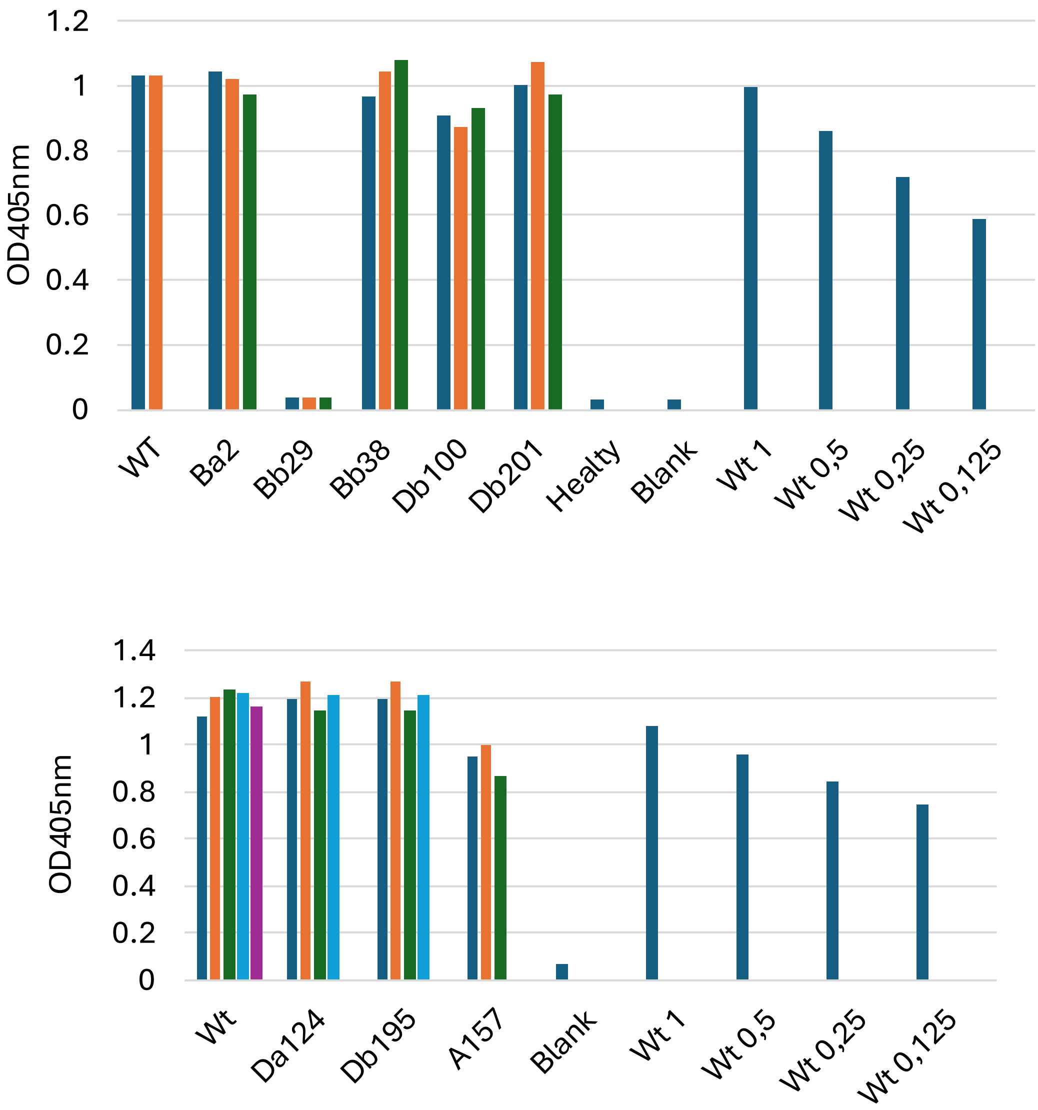

Figure S6. Screening for PVY resistance of Cas12a-edited potato plants. Wild-type and edited potato lines were challenged with PVY-Pa36, and virus accumulation was assessed between 21 and 30 days post-inoculation by double-antibody sandwich enzyme-linked immunosorbent assay. A pool of extracts from the wild-type PVY-Pa36-infected plants was used in a two-fold dilution series to assess virus accumulation.
