# Supplementary material for: Imprecise Cas12a/ssODN‐Mediated Editing of eIF4E1 Confers Dominant‐Negative Resistance to Potato Virus Y in Solanum tuberosum
Source: Mol Plant Pathol. 2026 Jun 30;27(7):e70305. doi: 10.1111/mpp.70305 (PMC13315812; doi:10.1111/mpp.70305)
Supplement: Supplementary file 7 — Figure S7: Hypersensitive response elicited by PVY‐O on the inoculated leaves of wild‐type and Bb29 Désirée plants. Two consecutive leaves of each plant were inoculated with the virus, L and U for lower and upper, respectively. The images were captured at the time reported on the left; dpi, days post‐inoculation. [file MPP-27-e70305-s003.pdf]

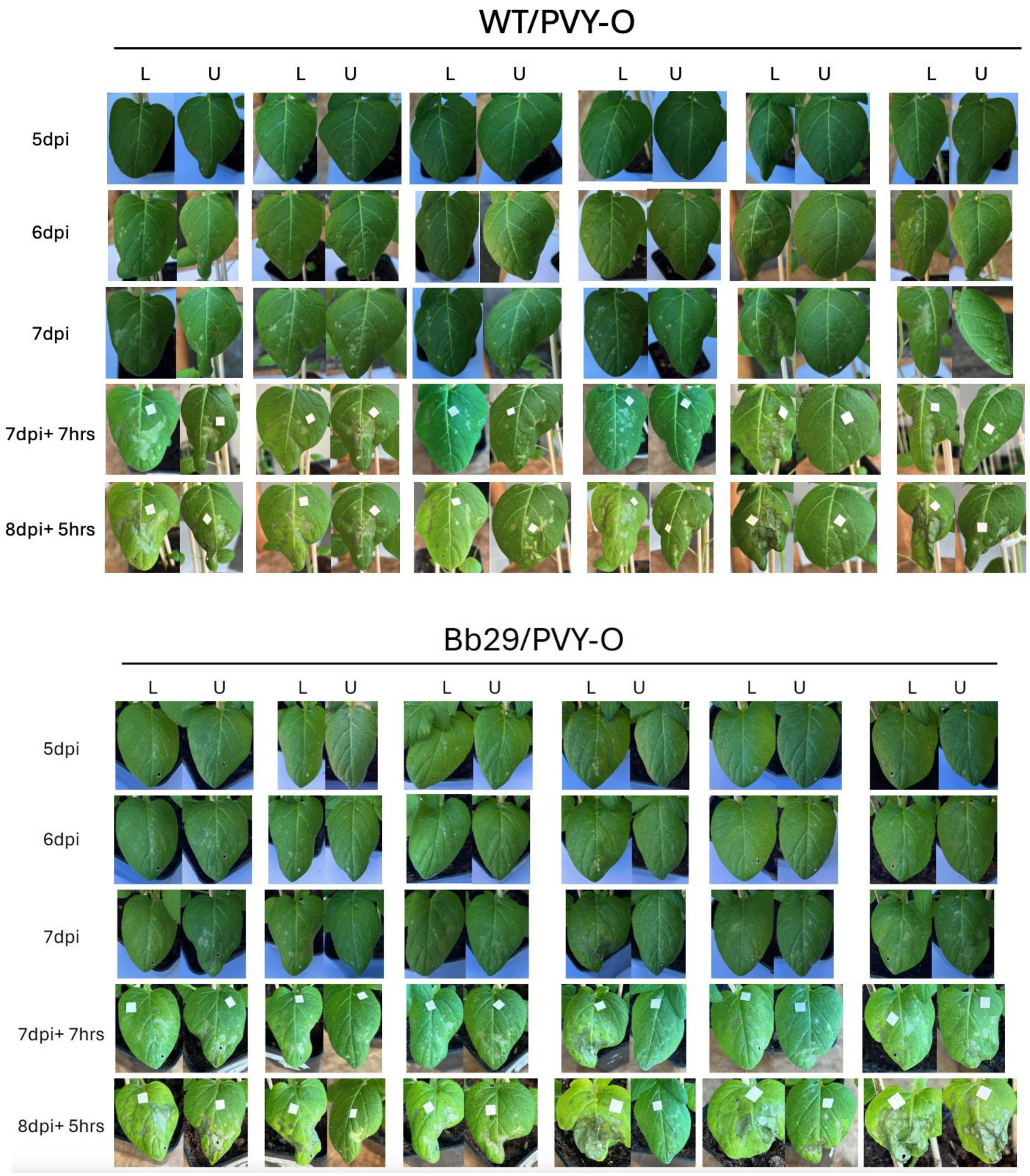

Figure S7. Hypersensitive response elicited by PVY-O on the inoculated leaves of wild-type and Bb29 Désirée plants. Two consecutive leaves of each plant were inoculated with the virus, L and U for lower and upper, respectively. The images were captured at the time reported on the left; dpi, days post inoculation.
