# Supplementary material for: Imprecise Cas12a/ssODN‐Mediated Editing of eIF4E1 Confers Dominant‐Negative Resistance to Potato Virus Y in Solanum tuberosum
Source: Mol Plant Pathol. 2026 Jun 30;27(7):e70305. doi: 10.1111/mpp.70305 (PMC13315812; doi:10.1111/mpp.70305)
Supplement: Supplementary file 8 — Figure S8: PVY‐O‐induced systemic necrosis in wild‐type (WT) Désirée potatoes is markedly reduced in Bb29 plants. (A) Comparison of WT and Bb29 plants at 14 dpi. Extended leaf necrosis develops in WT plants, while occasionally single lesions may occur in some Bb29 plants. Ctrl, uninoculated control WT plant. (B) Virus accumulation in systemic plant tissues of WT and Bb29 plants at 14 days post‐inoculation (dpi). A pool of extracts from the four WT plants was used in a two‐fold dilution series to assess virus accumulation. [file MPP-27-e70305-s005.pdf]

A

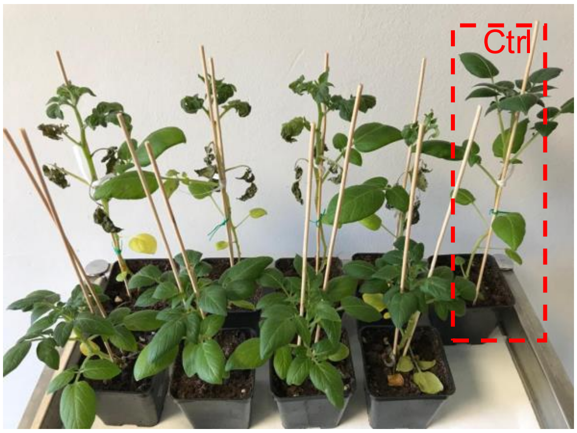

B

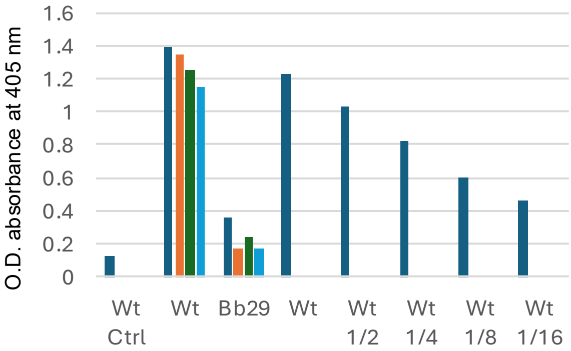

Figure S8. PVY-O-induced systemic necrosis in wild-type Désirée potatoes is markedly reduced in Bb29 plants.

A) Comparison of WT and Bb29 plants at 14 dpi. Extended leaf necrosis develops in WT plants, while occasionally single lesions may occur in some Bb29 plants. Ctrl, uninoculated control WT plant.

B) Virus accumulation in systemic plant tissues of WT and Bb29 plants at 14 days post-inoculation (dpi). A pool of extracts from the four WT plants was utilized in a two-fold dilution series to assess virus accumulation.
