# Supplementary material for: Imprecise Cas12a/ssODN‐Mediated Editing of eIF4E1 Confers Dominant‐Negative Resistance to Potato Virus Y in Solanum tuberosum
Source: Mol Plant Pathol. 2026 Jun 30;27(7):e70305. doi: 10.1111/mpp.70305 (PMC13315812; doi:10.1111/mpp.70305)
Supplement: Supplementary file 9 — Figure S9: Resistance analysis of Bb29 plants challenged with the resistance breaking PVY‐Pa36(K105E) isolate. Virus accumulation was assed at 21 days post‐inoculation by double‐antibody sandwish‐ELISA. Centre lines show the medians; box limits indicate the 25th and 75th percentiles, as determined by R. The numbers above the boxes indicate the number of plants analysed. [file MPP-27-e70305-s011.pdf]

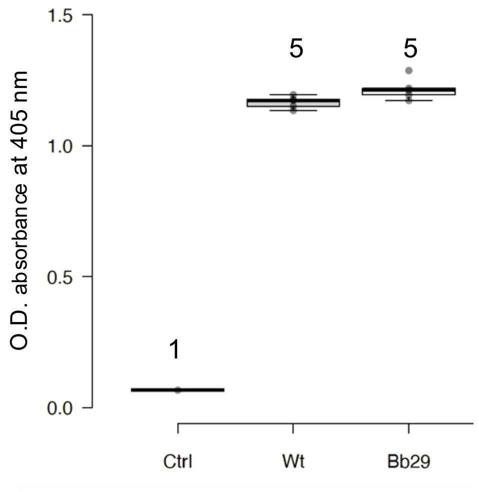


**Figure S9**. Resistance analysis of Bb29 plants challenged with the resistance breaking PVY-Pa36(K105E) isolate. Virus accumulation was assed at 21 dpi by DAS-ELISA. Center lines show the medians; box limits indicate the 25th and 75th percentiles, as determined by R. The numbers above the boxes indicate the number of plants analyzed.
