# Supplementary material for: Imprecise Cas12a/ssODN‐Mediated Editing of eIF4E1 Confers Dominant‐Negative Resistance to Potato Virus Y in Solanum tuberosum
Source: Mol Plant Pathol. 2026 Jun 30;27(7):e70305. doi: 10.1111/mpp.70305 (PMC13315812; doi:10.1111/mpp.70305)
Supplement: Supplementary file 10 — Figure S10: Inverse PCR for sequencing Bb29 eIF4E1_B1 mutated allele. (A) Schematic representation of the Bb29 genomic region surrounding exon 1 of the B1 allele. (B) Schematic representation as in (A), after HincII digestion and intramolecular ligation. Arrows represent primers used for PCR amplification and DNA sequencing. In the sequence below, red lowercase letters represent mutated nucleotides mimicking pvr2 1 , and light blue, italicized duplicated sequences. (C) Sequencing results of the PCR fragment obtained using primers indicated. [file MPP-27-e70305-s001.pdf]

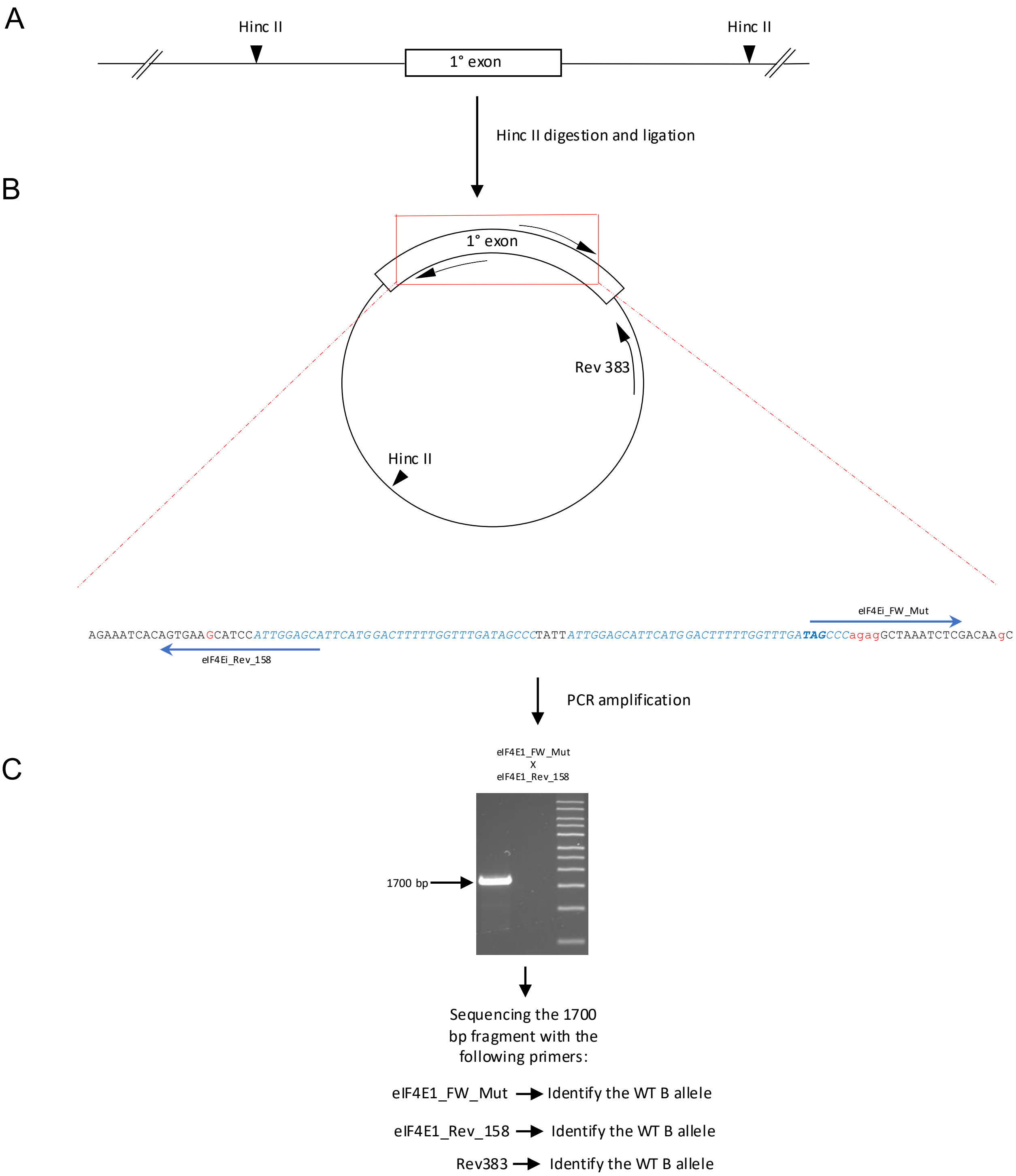

Figure S10. Inverse PCR for sequencing Bb29 *eIF4E1_B1* mutated allele

A) Schematic representation of the Bb29 genomic region surrounding exon 1 of the B1 allele
B) Schematic representation as in (A), after *Hinc*II digestion and intramolecular ligation. Arrows represent primers used for PCR amplification and DNA sequencing. In the sequence below, red lowercase letters represent mutated nucleotides mimicking *pvr2*[1], and light blue, italicized duplicated sequences.
C) Sequencing results of the PCR fragment obtained using primers indicated.
