# Supplementary material for: Imprecise Cas12a/ssODN‐Mediated Editing of eIF4E1 Confers Dominant‐Negative Resistance to Potato Virus Y in Solanum tuberosum
Source: Mol Plant Pathol. 2026 Jun 30;27(7):e70305. doi: 10.1111/mpp.70305 (PMC13315812; doi:10.1111/mpp.70305)
Supplement: Supplementary file 11 — Figure S11: Yeast complementation analyses of SteIF4E1_A and SteIF4E1_B mutated alleles of Bb29 potato plants. (A) Amino acid sequences of the region I (Poulicard et al. 2016) of SteIF4E1 proteins. SteIF4E1_A and SteIF4E1_B wild‐type proteins; SteIF4E1_AΔ12 and SteIF4E1_BΔ6 proteins derived from SteIF4E1_A and B Bb29 mutated alleles. Deleted amino acids are represented with en‐dashes. (B) The yeast strain JO55 was transformed with either an empty p424GPD plasmid (negative control) or with p424GPD constructs expressing SteIF4E1_A and SteIF4E1_B (positive controls) and Bb29 mutated SteIF4E1_AΔ12 and SteIF4E1_BΔ6 alleles. Dilutions were spotted on galactose/raffinose (Gal/Raf) medium and on a selective medium containing glucose. Only yeast functionally complemented by the ectopic expression of an eIF4E protein can grow on the selective medium. (C) Western blot of protein extracts from yeast strain JO55 transformed with (1) p424GPD_SteIF4E1_A; (2 and 4) p424GPD_SteIF4E1_B; (3) p424GPD empty vector; (5) p424GPD_SteIF4E1_AΔ12; (6) p424GPD_SteIF4E1_BΔ6. Yeast was grown on galactose and raffinose as carbon sources, except for yeast in line 4, which was grown on glucose. The lower panel shows Coomassie staining of a replica gel to assess protein loading. [file MPP-27-e70305-s006.pdf]

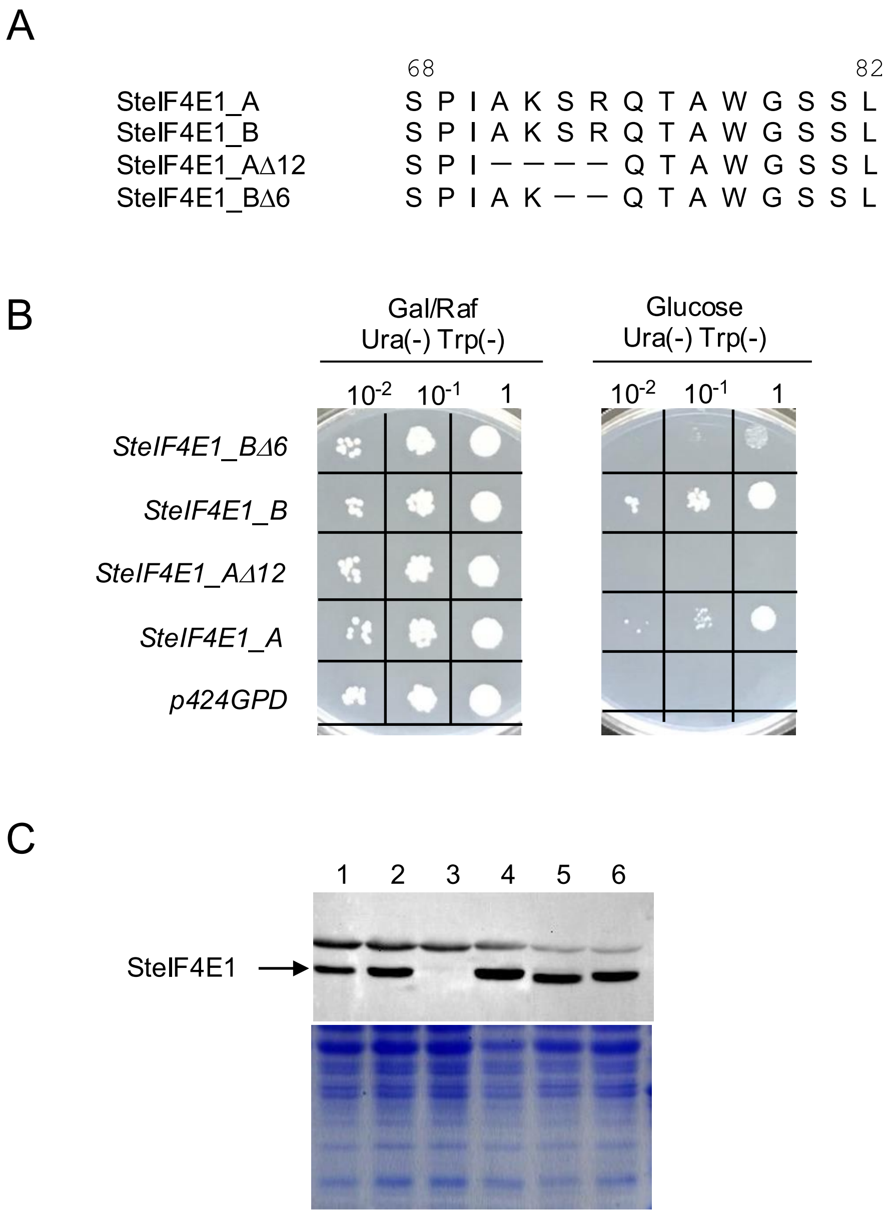

Figure S11. Yeast complementation analyses of *SteIF4E1_A* and *SteIF4E1_B* mutated alleles of Bb29 potato plants.

(A) Amino acid sequences of the region I (Poulicard et al., 2016) of SteIF4E1 proteins. SteIF4E1_A and SteIF4E1_B wild-type proteins; SteIF4E1_AΔ12 and SteIF4E1_BΔ6 proteins derived from *SteIF4E1_A* and *B Bb29* mutated alleles. Deleted amino acids are represented with en-dashes.

(B) The yeast strain JO55 was transformed with either an empty p424GPD plasmid (negative control) or with p424GPD constructs expressing *SteIF4E1_A* and *SteIF4E1_B* (positive controls) and Bb29 mutated *SteIF4E1_AΔ12* and *SteIF4E1_BΔ6* alleles. Dilutions were spotted on galactose/raffinose (Gal/Raf) medium and on a selective medium containing glucose. Only yeast functionally complemented by the ectopic expression of an eIF4E protein can grow on the selective medium.

(C) Western blot of protein extracts from yeast strain JO55 transformed with: (1) p424GPD_*SteIF4E1_A*; (2 and 4) p424GPD_*SteIF4E1_B*; (3) p424GPD empty vector; (5) p424GPD_*SteIF4E1_AΔ12*; (6) p424GPD_*SteIF4E1_BΔ6*. Yeast was grown on galactose and raffinose as carbon sources, except for yeast in line 4, which was grown on glucose. The lower panel shows Coomassie staining of a replica gel to assess protein loading.
