# Supplementary material for: Imprecise Cas12a/ssODN‐Mediated Editing of eIF4E1 Confers Dominant‐Negative Resistance to Potato Virus Y in Solanum tuberosum
Source: Mol Plant Pathol. 2026 Jun 30;27(7):e70305. doi: 10.1111/mpp.70305 (PMC13315812; doi:10.1111/mpp.70305)
Supplement: Supplementary file 12 — Figure S12: Sequence analysis of eIF4E1 alleles containing pvr21SD mutations. For each edited line, the mutated sequence is aligned with the theoretical mutated sequence. Red letters: mutated nucleotides in the ssODN. Duplicated sequences are underlined and identified as x1 and x2 (with × spanning from A to M) and the x2 sequence is in light blue. When the duplicated sequence is in the inverted orientation, the repeated inverted sequence is in light blue italic. Boxed sequences: Cas12a PAM site. Bold letters: stop codon. [file MPP-27-e70305-s015.pdf]

Figure S12

A38_A

```
                                                 A1
A38_A      AGAAGGTGAAATTGTTGAAGAATCAAATGATACGGCGTCGTATTTAGGGAAAGAAATCAC 60
Amut       AGAAGGTGAAATTGTTGAAGAATCAAATGATACGGCGTCGTATTTAGGGAAAGAAATCAC 60
           ************************************************************

A38_A      AGTGAAACATCCATTGGAGCATTCATGGACTTTTTGGTTTGATAGCCCTATTGCTATCAT 120
Amut       AGTGAAACATCCATTGGAGCATTCATGGACTTTTTGGTTTGATAGCCC------------ 108
           ************************************************

                   A2                                B1             B2
A38_A      GGACTTTTTGGTTTTGATAGCCCAGAGGCTAAATCTCGACAAGCTGCTTGTCGACAAACT 180
Amut       -----------------------AGAGGCTAAATCTCGACAAGCTGCTT----------- 134
                                  **************************

A38_A      GCTTGGGGAAGCTCACTTCGAAATGTCTACACTTTCTCCACTGTTGAAGATTTTTGGGGG 240
Amut       ----GGGGAAGCTCAAGACGAAATGTCTACACTTTCTCCACTGTTGAAGATTTTTGGGGG 190
               ***********   ******************************************

A38_A      TAATTTTTTTTTCTTTCAAATTGGTGATAGTGTAGTGTAAGAGGAAACAGGGGAATGGAT 300
Amut       TAATTTTTTTTTCTTTCAAATTGGTGATAGTGTAGTGTAAGAGGAAACAGGGGAATGGAT 250
           ************************************************************

A38_A      CGCAAGGTGTGGAATCGAATCCTCCTGAAAGTTTAGATAGTCAATTAATTGAGCTACTGA 360
Amut       CGCAAGGTGTGGAATCGAATCCTCCTGAAAGTTTAGATAGTCAATTAATTGAGCTACTGA 310
           ************************************************************

A38_A      GATTCCCCGGAT  372
Amut       GATTCCCCGGAT  322
           ************
```

Ba13_B1

```
Ba13_B1      AGAAGGTGAAATTGTTGAAGAATCAAATGATACGGCGTCGTATTTAGGGAAAGAAATCAC    60
B1mut        AGAAGGTGAAATTGTTGAAGAATCAAATGATACGGCGTCGTATTTAGGGAAAGAAATCAC    60
             ************************************************************

                                                       C1               C2
Ba13_B1      AGTGAAGCATCCATTGGAGCATTCATGGACTTTTTGGTTTGATAGCCCTATTTTGTTTGA    120
B1mut        AGTGAAGCATCCATTGGAGCATTCATGGACTTTTTGGTTTGATAGCCC------------    107
             ************************************************

                                                           D1
Ba13_B1      TAGCCCAGAGGCTAAATCTCGACAAGCTGCTTGGGGAAGCTCAAGACGAAATGTCTACAC    180
B1mut        ------AGAGGCTAAATCTCGACAAGCTGCTTGGGGAAGCTCCAGACGAAATGTCTACAC    123
                   ************************************ *****************

                                                          D2
Ba13_B1      TTTCTCCACTGTCGAGATTTAGCCAAACTGCTTGGGGAAGCTCCCTTCGAAATGTCTACA    240
B1mut        ------------------------------------------------------------    161

Ba13_B1      CTTTCTCCACTGTTGAAGATTTTTGGGGGTAATTTTTTTTTTCTTTCAAATTGGTGCTAG    300
B1mut        -TTTCTCCACTGTTGAAGATTTTTGGGGGTAATTTTTTTTTTCTTTCAAATTGGTGCTAG    221
              ***********************************************************

Ba13_B1      TGTAGTGTAAGAGGAAACAGGGGGAGTGGATCGCAAGGTGTGGAATCGAATCCTCGTGAA    360
```

```
B1mut         TGTAGTGTAAGAGGAAACAGGGGGAGTGGATCGCAAGGTGTGGAATCGAATCCTCGTGAA    281
              ************************************************************

Ba13_B1       AGTTTAGATAGTCAATTAATTGAGCTACTGAGATTCCCCGGAT   403
B1mut         AGTTTAGATAGTCAATTAATTGAGCTACTGAGATTCCCCGGAT   324
              *******************************************
```

Ba44_B

```
Ba44_B       AGAAGGTGAAATTGTTGAAGAATCAAATGATATGGCGTCGTATTTAGGGAAAGAAATCAC     60
Bmut         AGAAGGTGAAATTGTTGAAGAATCAAATGATATGGCGTCGTATTTAGGGAAAGAAATCAC     60
             ************************************************************

                                                        D1          D2
Ba44_B       AGTGAAGCATCCATTGGAGCATTCATGGACTTTTTGGTTTGATAGCCCTATTGCTAGCCC     120
Bmut         AGTGAAGCATCCATTGGAGCATTCATGGACTTTTTGGTTTGATAGCCC------------     118
             ************************************************

                            E1  E2
Ba44_B       AGAGGCTAAATCTTCGACGACAAACTGCTTGGGGAAGCTCACTTCGAAATGTCTACACTT     180
Bmut         AGAGGCTAAATCT----CGACAAGCTGCTTGGGGAAGCTCAAGACGAAATGTCTACACTT     164
             *************    ****** *****************   ****************

Ba44_B       TCTCCACTGTTGAAGATTTTTGGGGGTAATTTTTTTTTTTCAAATTGGTGATAGTGTAGT     240
Bmut         TCTCCACTGTTGAAGATTTTTGGGGGTAATTTTTTTTTTTCAAATTGGTGATAGTGTAGT     224
             ************************************************************

Ba44_B       GTAAGAGGAAACAGGGGAGTGGATCGCAAGGTGTGGAAACGAATCCTCCTGAAAGTTTAG     300
Bmut         GTAAGAGGAAACAGGGGAGTGGATCGCAAGGTGTGGAAACGAATCCTCCTGAAAGTTTAG     284
             ************************************************************

Ba44_B       ATAGTCAATTAATTGAGCTACTGAGATTCCCCGGAT     336
Bmut         ATAGTCAATTAATTGAGCTACTGAGATTCCCCGGAT     320
             ************************************
```

DB100_B1

```
DB100_B1 AGAAGGTGAAATTGTTGAAGAATCAAATGATACGGCGTCGTATTTAGGGAAAGAAATCAC 60
B1mut    AGAAGGTGAAATTGTTGAAGAATCAAATGATACGGCGTCGTATTTAGGGAAAGAAATCAC 60
         ************************************************************

                                                    F1           F2
DB100_B1 AGTGAAGCATCCATTGGAGCATTCATGGACTTTTTGGTTTGATAGCCCTATTGCTTGATA 120
B1mut    AGTGAAGCATCCATTGGAGCATTCATGGACTTTTTGGTTTGATAGCCC------------ 108
         ************************************************
                             G1       G2
DB100_B1 GCCCAGAGGCTAAATCTCGACAAGCTGACAAACTGCTTGGGGAAGCTCCCTTCGAAATGT 180
B1mut    ----AGAGGCTAAATCTCGACAAGCTG--------CTTGGGGAAGCTCCAGACGAAATGT 156
             ***********************        **************   ********

DB100_B1 CTACACTTTCTCCACTGTTGAAGATTTTTGGGGGTAATTTTTTTTTTCTTTCAAATTGGT 240
B1mut    CTACACTTTCTCCACTGTTGAAGATTTTTGGGGGTAATTTTTTTTTTCTTTCAAATTGGT 216
         ************************************************************

DB100_B1 GCTAGTGTAGTGTAAGAGGAAACAGGGGGAGTGGATCGCAAGGTGTGGAATCGAATCCTC 300
B1mut    GCTAGTGTAGTGTAAGAGGAAACAGGGGGAGTGGATCGCAAGGTGTGGAATCGAATCCTC 276
         ************************************************************

DB100_B1 GTGAAAGTTTAGATAGTCAATTAATTGAGCTACTGAGATTCCCCGGAT 348
```

```
B1mut      GTGAAAGTTTAGATAGTCAATTAATTGAGCTACTGAGATTCCCCGGAT 324
           ************************************************
```

DB190_B1

```
DB190_B1     AGAAGGTGAAATTGTTGAAGAATCAAATGATACGGCGTCGTATTTAGGGAAAGAAATCAC  60
B1mut        AGAAGGTGAAATTGTTGAAGAATCAAATGATACGGCGTCGTATTTAGGGAAAGAAATCAC  60
             ************************************************************

                                                        F1            F2
DB190_B1     AGTGAAGCATCCATTGGAGCATTCATGGACTTTTTGGTTTGATAGCCCTATTGCTTGATA  120
B1mut        AGTGAAGCATCCATTGGAGCATTCATGGACTTTTTGGTTTGATAGCCC------------  108
             ************************************************

                                       G1       G2
DB190_B1     GCCCAGAGGCTAAATCTCGACAAGCTGACAAACTGCTTGGGGAAGCTCCCTTCGAAATGT  180
B1mut        ----AGAGGCTAAATCTCGACAAGCTG--------CTTGGGGAAGCTCCAGACGAAATGT  156
                 ***********************        ***************   *******

DB190_B1     CTACACTTTCTCCACTGTTGAAGATTTTTGGGGGTAATTTTTTTTTTCTTTCAAATTGGT  240
B1mut        CTACACTTTCTCCACTGTTGAAGATTTTTGGGGGTAATTTTTTTTTTCTTTCAAATTGGT  216
             ************************************************************

DB190_B1     GCTAGTGTAGTGTAAGAGGAAACAGGGGGAGTGGATCGCAAGGTGTGGAATCGAATCCTC  300
B1mut        GCTAGTGTAGTGTAAGAGGAAACAGGGGGAGTGGATCGCAAGGTGTGGAATCGAATCCTC  276
             ************************************************************

DB190_B1     GTGAAAGTTTAGATAGTCAATTAATTGAGCTACTGAGATTCCCCGGAT  348
B1mut        GTGAAAGTTTAGATAGTCAATTAATTGAGCTACTGAGATTCCCCGGAT  324
             ************************************************
```

Ba2_A

```
Ba2_A        AGAAGGTGAAATTGTTGAAGAATCAAATGATACGGCGTCGTATTTAGGGAAAGAAATCAC     60
Amut         AGAAGGTGAAATTGTTGAAGAATCAAATGATACGGCGTCGTATTTAGGGAAAGAAATCAC     60
             ************************************************************

                                                       H1
Ba2_A        AGTGAAACATCCATTGGAGCATTCATGGACTTTTTGGTTTGATAGCCCTATTGCTATTTT     120
Amut         AGTGAAACATCCATTGGAGCATTCATGGACTTTTTGGTTTGATAGCCC------------     112
             ************************************************

                       H2
Ba2_A        GGTTTGATAGCCCAGAGGCTAAATCTCGACAAGCGGCTTGGGGAAGCTCACTTCGAAATG     180
Amut         -------------AGAGGCTAAATCTCGACAAGCTGCTTGGGGAAGCTCAAGACGAAATG     155
                          ******************** ***************   *******

Ba2_A        TCTACACTTTCTCCACTGTTGAAGATTTTTGGGGGTAATTTTTTTTTTCTTTCAAATTGGT    240
Amut         TCTACACTTTCTCCACTGTTGAAGATTTTTGGGGGTAATTTTTTTTTTCTTTCAAATTGGT    215
             ************************************************************

Ba2_A        GATAGTGTAGTGTAAGAGGAAACAGGGGAATGGATCGCAAGGTGTGGAATCGAATCCTCC     300
Amut         GATAGTGTAGTGTAAGAGGAAACAGGGGAATGGATCGCAAGGTGTGGAATCGAATCCTCC     275
             ************************************************************

Ba2_A        TGAAAGTTTAGATAGTCAATTAATTGAGCTACTGAGATTCCCCGGAT 347
Amut         TGAAAGTTTAGATAGTCAATTAATTGAGCTACTGAGATTCCCCGGAT 322
             ***********************************************
```

Ba25_B

```
                                                                              
Ba25_B      AGAAGGT-AAATTGTTGAAGAATCAAATGATATGGCGTCGTATTTAGGGAAAGAAATCAC     59
Bmut        AGAAGGTGAAATTGTTGAAGAATCAAATGATATGGCGTCGTATTTAGGGAAAGAAATCAC     60
            ******* ****************************************************

                                                       I1
Ba25_B      AGTGAAGCATCCATTGGAGCATTCATGGACTTTTTGGTTTGATAGCCCTATTGCTACTCG     119
Bmut        AGTGAAGCATCCATTGGAGCATTCATGGACTTTTTGGTTTGATAGCCCAGAGGCT-----     115
            ************************************************    ***

              ______ Rev L1________ _________Rev I1_____________        L1
Ba25_B      ACAAGCAGCTTGTCGAGATTTAGCCTCTGGGCTATCAAACCAAAAAAGTCGTCCCGACAA     179
Bmut        -------------------------------------------------AAATCTCGACAA    127
                                                             ** ******

              L1
Ba25_B      GCTGCTTGGGGAAGCTCACTTCGAAATGTCTACACTTTCTCCACTGTTGAAGATTTTTGG     239
Bmut        GCTGCTTGGGGAAGCTCAAGACGAAATGTCTACACTTTCTCCACTGTTGAAGATTTTTGG     187
            ******************   ***************************************

Ba25_B      GGGTAATTTTTTTTTTTCAAATTGGTGATAGTGTAGTGTAAGAGGAAACAGGGGAGTGGA     299
Bmut        GGGTAATTTTTTTTTTTCAAATTGGTGATAGTGTAGTGTAAGAGGAAACAGGGGAGTGGA     247
            ************************************************************

Ba25_B      TCGCAAGGTGTGGAAACGAATCCTCCTGAAAGTTTAGATAGTCAATTAATTGAGCTACTG     359
Bmut        TCGCAAGGTGTGGAAACGAATCCTCCTGAAAGTTTAGATAGTCAATTAATTGAGCTACTG     307
            ************************************************************

Ba25_B      AGATTCCCCGGAT      372
Bmut        AGATTCCCCGGAT      320
            *************
```

Da118_B

```
Da118_B      AGAAGGTGAAATTGTTGAAGAATCAAATGATATGGCGTCGTATTTAGGGAAAGAAATCAC    60
Bmut         AGAAGGTGAAATTGTTGAAGAATCAAATGATATGGCGTCGTATTTAGGGAAAGAAATCAC    60
             ************************************************************

Da118_B      AGTGAAGCATCCATTGGAGCATTCATGGACTTTTTGGTTTGATAGCCCTATTGCTTTCGT    120
Bmut         AGTGAAGCATCCATTGGAGCATTCATGGACTTTTTGGTTTGATAGCCCAGAGGCT-----    111
             ************************************************    ***

                  RevM1                                     M1
Da118_B      CTTGAGCTTCCCCAAGCAGCTTCTCGACAAACTGCTTGGGGAAGCTCACTTCGAAATGTC    180
Bmut         ------------------AAATCTCGACAAGCTGCTTGGGGAAGCTCAAGACGAAATGTC    157
                                 ********* *****************   *********

Da118_B      TACACTTTCTCCACTGTTGAAGATTTTTGGGGGTAATTTTTTTTTTTCAAATTGGTGATA    240
Bmut         TACACTTTCTCCACTGTTGAAGATTTTTGGGGGTAATTTTTTTTTTTCAAATTGGTGATA    217
             ************************************************************

Da118_B      GTGTAGTGTAAGAGGAAACAGGGGAGTGGATCGCAAGGTGTGGAAACGAATCCTCCTGAA    300
Bmut         GTGTAGTGTAAGAGGAAACAGGGGAGTGGATCGCAAGGTGTGGAAACGAATCCTCCTGAA    277
             ************************************************************

Da118_B      AGTTTAGATAGTCAATTAANTGAGCTACTGAGATTCCCCGGAT      343
Bmut         AGTTTAGATAGTCAATTAATTGAGCTACTGAGATTCCCCGGAT      320
             ******************* ***********************
```

Figure S12. Sequence analysis of eIF4E1 alleles containing pvr2[1]SD mutations.
For each edited line, the mutated sequence is aligned with the theoretical mutated sequence. Red letters: mutated nucleodites in the ssODN. Duplicated sequences are underlined and identified as x1 and x2 (with x spanning from A to

M) and the x2 sequence is in light blue. When the duplicated sequence is in the inverted orientation, the repeated inverted sequence is in light blue italic. Boxed sequences: Cas12a PAM site. Bold letters: stop codon.
