# Supplementary material for: Imprecise Cas12a/ssODN‐Mediated Editing of eIF4E1 Confers Dominant‐Negative Resistance to Potato Virus Y in Solanum tuberosum
Source: Mol Plant Pathol. 2026 Jun 30;27(7):e70305. doi: 10.1111/mpp.70305 (PMC13315812; doi:10.1111/mpp.70305)
Supplement: Supplementary file 13 — Figure S13: Height analysis of uninoculated Bb29 plants transformed with SteIF4E1_A allele (A lines), SteIF4E1_B allele (B lines) or an empty vector (V lines) as a control. (A). The label DN stands for the wild‐type Désirée. Plants were photographed at time zero when the other plants in the pair were inoculated with PVY‐Pa36. (B) Plants at 21 days after time zero. [file MPP-27-e70305-s013.pdf]

A

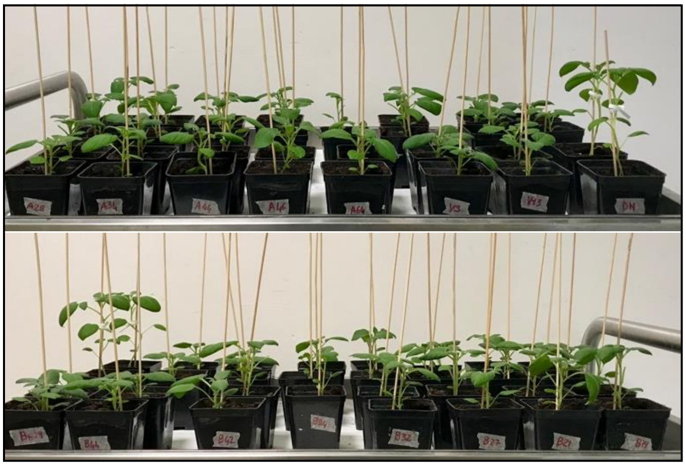

B

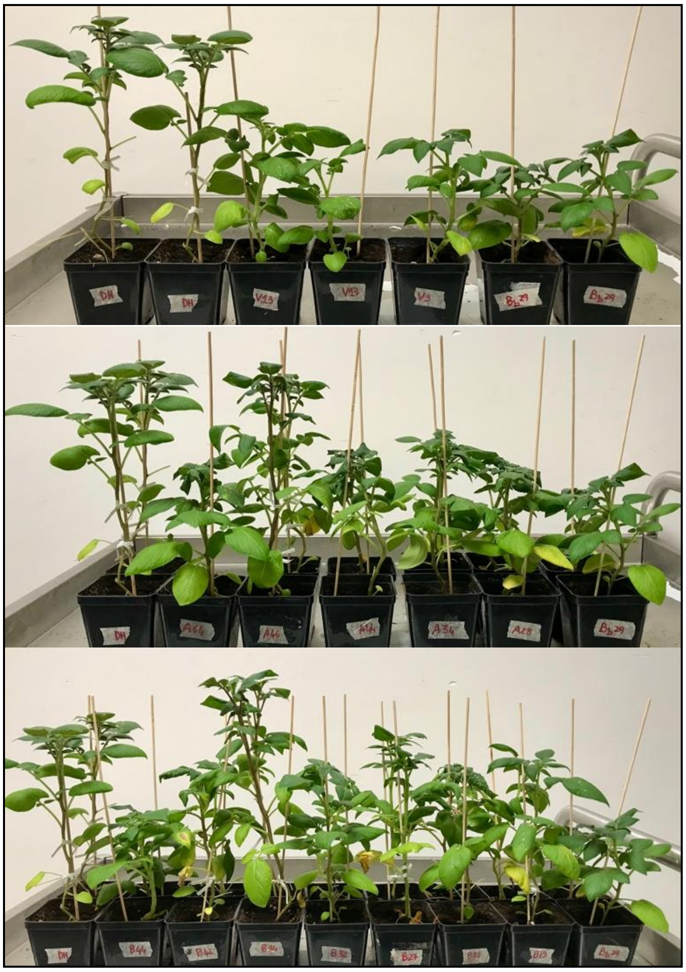

Figure S13. Height analysis of uninoculated Bb29 plants transformed with *SteIF4E1_A* allele (A lines), *SteIF4E1_B* allele (B lines), or an empty vector (V lines) as a control. A). The label DN stands for the WT Désirée. Plants were photographed at time zero when the other plants in the pair were inoculated with PVY-Pa36. B) Plants at 21 days after time zero.
