# Supplementary material for: Imprecise Cas12a/ssODN‐Mediated Editing of eIF4E1 Confers Dominant‐Negative Resistance to Potato Virus Y in Solanum tuberosum
Source: Mol Plant Pathol. 2026 Jun 30;27(7):e70305. doi: 10.1111/mpp.70305 (PMC13315812; doi:10.1111/mpp.70305)
Supplement: Supplementary file 14 — Figure S14: PVY‐Pa36 resistance analyses of wild‐type Désirée plants transformed with the Bb29 mutated SteIF4E1_AΔ12 allele. Virus accumulation was evaluated at 30 days post‐inoculation by double‐antibody sandwich‐ELISA. D12‐4, D12‐17, D12‐42, D12‐59, D12‐98 transgenic lines for the SteIF4E1_AΔ12 allele. P7 or P12 transgenic lines for the empty vector. Control, non‐inoculated P7 or P12 plant. The sap from P7 or P12 plants was mixed and used to generate the standard curve. [file MPP-27-e70305-s017.pdf]

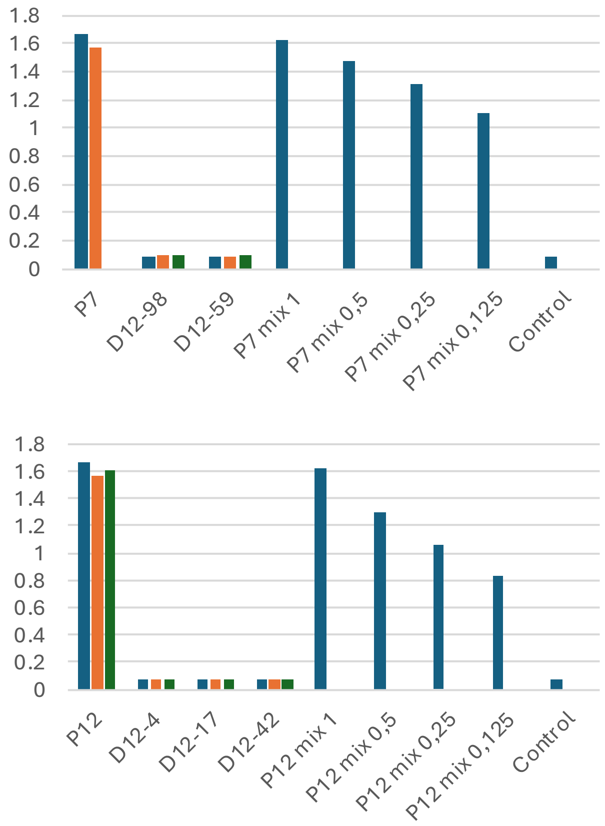

Figure S14. PVY-Pa36 resistance analyses of WT Désirée plants transformed with the Bb29 mutated *StelF4E1_AΔ12* allele. Virus accumulation was evaluated at 30 days post-inoculation by DAS-ELISA. D12-4, D12-17, D12-42, D12-59, D12-98 transgenic lines for the *StelF4E1_AΔ12* allele. P7 or P12 transgenic lines for the empty vector.
Control, non-inoculated P7 or P12 plant. The sap from P7 or P12 plants was mixed and used to generate the standard curve.
