# Supplementary material for: Imprecise Cas12a/ssODN‐Mediated Editing of eIF4E1 Confers Dominant‐Negative Resistance to Potato Virus Y in Solanum tuberosum
Source: Mol Plant Pathol. 2026 Jun 30;27(7):e70305. doi: 10.1111/mpp.70305 (PMC13315812; doi:10.1111/mpp.70305)
Supplement: Supplementary file 15 — Table S1: mpp70305‐sup‐0015‐TableS1.docx. SteIF4E1_pvr2 1 potato editing. [file MPP-27-e70305-s002.docx]

**Table S1**. *SteIF4E1_pvr2^1^* potato editing

| **Transfection experiments** | **Regenerated plants** | **^a^PCR-positive plants** | **% of PCR-positive plants** |
| --- | --- | --- | --- |
| ^b^A | 197 | 4 | 2 |
| ^b^Ba | 65 | 16 | 25 |
| ^b^Bb | 42 | 8 | 19 |
| ^c^Da | 128 | 7 | 5 |
| ^c^Db | 201 | 10 | 5 |
| A+Ba+Bb+Da+Db | 633 | 45 | 7.1 |

^a^Oligos used in the screening, eIF4e_LEI_Fw20 x eIF4e_Con_Rev

^b^Potato protoplasts transfected with the mutated ssODN and the Cas12a/crRNA ribonucleoprotein complex

^c^Potato protoplasts transfected with the mutated ssODN and the plasmid encoding Cas12a and the crRNA.
